# Updating the Quarantine Status of *Prunus* Infecting Viruses in Australia

**DOI:** 10.3390/v12020246

**Published:** 2020-02-23

**Authors:** Wycliff M. Kinoti, Narelle Nancarrow, Alison Dann, Brendan C. Rodoni, Fiona E. Constable

**Affiliations:** 1 Agriculture Victoria, AgriBio, Centre for AgriBioscience, Bundoora, VIC 3083, Australia; 2 Agriculture Victoria, Horsham, VIC 3400, Australia; 3 Plant Biosecurity and Diagnostic Branch, Bioisecurity Tasmania, Hobart, TAS 7001, Australia

**Keywords:** *Prunus*, viruses, metagenomic high-throughput sequencing

## Abstract

One hundred *Prunus* trees, including almond (*P. dulcis*), apricot (*P. armeniaca*), nectarine (*P. persica var. nucipersica*), peach (*P. persica*), plum (*P. domestica*), purple leaf plum (*P. cerasifera*) and sweet cherry (*P. avium*), were selected from growing regions Australia-wide and tested for the presence of 34 viruses and three viroids using species-specific reverse transcription-polymerase chain reaction (RT-PCR) or polymerase chain reaction (PCR) tests. In addition, the samples were tested using some virus family or genus-based RT-PCR tests. The following viruses were detected: *Apple chlorotic leaf spot virus* (ACLSV) (13/100), *Apple mosaic virus* (ApMV) (1/100), *Cherry green ring mottle virus* (CGRMV) (4/100), *Cherry necrotic rusty mottle virus* (CNRMV) (2/100), *Cherry virus A* (CVA) (14/100), *Little cherry virus 2* (LChV2) (3/100), *Plum bark necrosis stem pitting associated virus* (PBNSPaV) (4/100), *Prune dwarf virus* (PDV) (3/100), *Prunus necrotic ringspot virus* (PNRSV) (52/100), *Hop stunt viroid* (HSVd) (9/100) and *Peach latent mosaic viroid* (PLMVd) (6/100). The results showed that PNRSV is widespread in *Prunus* trees in Australia. Metagenomic high-throughput sequencing (HTS) and bioinformatics analysis were used to characterise the genomes of some viruses that were detected by RT-PCR tests and *Apricot latent virus* (ApLV), *Apricot vein clearing associated virus* (AVCaV), *Asian Prunus Virus 2* (APV2) and *Nectarine stem pitting-associated virus* (NSPaV) were also detected. This is the first report of ApLV, APV2, CGRMV, CNRNV, LChV1, LChV2, NSPaV and PBNSPaV occurring in Australia. It is also the first report of ASGV infecting *Prunus* species in Australia, although it is known to infect other plant species including pome fruit and citrus.

## 1. Introduction

Worldwide *Prunus* species, including *P. armeniaca* (apricot), *P. avium* (sweet cherry), *P. cerasifera* (purple leaf plum), *P. domestica* (plum), *P. dulcis* (almond), *P. persica* (peach) and *P. persica* var. *nucipersica* (nectarine), are commercially grown for their nuts, fruits and as ornamental plants [1,2,3]. Almond and stone fruit species, such as apricot, cherry, peach and plums, were first introduced into Australia during the 1800s and there have been many subsequent introductions to improve plant genetics and to support the development of commercial almond and stone fruit orchards in Australia [4,5,6]. Currently, the almond and stone fruit industries form one of the largest and most rapidly expanding agricultural industries in Australia [7,8] and the development of these industries continues to rely heavily on imported germplasm.

The biosecurity of the Australian almond and stone fruit industries are maintained at the border by the Department of Agriculture. *Prunus* germplasm imported into Australia requires a minimum of 18 months post-entry quarantine (PEQ) and during that time it is tested using a range of diagnostic techniques for the detection of diseases and pathogens of quarantine significance. Post-border, the biosecurity of these industries is maintained through certification schemes that supply high-health planting material throughout Australia and which, until recently, was tested only for the virus species *Apple chlorotic leaf spot virus* (ACLSV), genus *Trichovirus,* and three species of the genus *Ilarvirus*: *Apple mosaic virus* (ApMV), *Prune dwarf virus* (PDV) and *Prunus necrotic ringspot virus* (PNRSV) [9,10].

Significant advances in molecular and diagnostic technology, such as reverse transcription (RT)-polymerase chain reaction (PCR), PCR and high-throughput sequencing (HTS), have allowed characterisation of new and emerging pathogens and new strains of known pathogens of *Prunus* species. It has helped to clarify pathogens, especially viruses, associated with diseases in *Prunus* species that had an unknown aetiology. There are now 55 virus species and three viroid species that have been reported to infect almonds, stone fruit and/or ornamental *Prunus* trees worldwide [11,12,13,14,15,16,17,18,19,20,21,22,23,24,25,26,27,28].

Due to the discovery of new and novel viruses and virus strains in *Prunus* species overseas and because there was little information about the incidence of viruses and viroids in Australian *Prunus* species, a national survey of the almond and stone fruit growing regions of Australia was conducted during 20 months in 2013–2015. The survey was used to update the disease status for viruses and viroids of almond and stone fruit industries in Australia and to update the list of viruses and viroids requiring testing during PEQ. The genomes of interesting Australian strains of *Prunus*-infecting viruses were assembled so that they were represented amongst other international isolates. In undertaking the survey, the molecular diagnostic tests for detection of these viruses were also validated. The results of the survey are reported here.

## 2. Materials and Methods

### 2.1. Sampling and Nucleic Acid Extraction

Four to eight shoots, with leaves attached, were randomly sampled per tree from 100 *Prunus* trees during spring and summer from August 2013 to March 2015 throughout Australia. They were collected from major almond and stone fruit growing regions and some home gardens (Table 1). Older varieties were selected in preference to more recent importations.

For each of the 100 *Prunus* trees, tissue from all shoots of an individual tree were subsampled and pooled for extraction. Total nucleic acid (TNA) was extracted from *Prunus* tree samples using a modified lysis buffer [29] and the QIAxtractor (QIAGEN) as described previously [30], except 200 µL of the combined sample lysate/ethanol mix was added to the filter plate instead of 500 µL. Three separate TNA extractions were carried out for each sample and these three extractions were combined to ensure that there was ample TNA for all the RT-PCR tests.

### 2.2. Virus and Viroids RT-PCR and PCR

The SuperScript™ One-Step RT-PCR System (Invitrogen, Scoresby, VIC, Australia) was used for detection of RNA viruses and viroids according to the manufacturer’s instructions except that 0.5 µL of SuperScript™ Taq enzyme mixture and 1 µL of template was used in a total RT-PCR reaction volume of 25 µL. A Platinum^®^ *Taq* DNA Polymerase (Invitrogen) kit was used for the nested PCR step of the genus or family-based tests (Table S1), according to the manufacturer’s instructions except that 0.2 µL of Platinum^®^ *Taq* DNA Polymerase and 1 µL of template was used in a total reaction volume of 25 µL.

Primers for the detection of NADH dehydrogenase ND2 subunit (*ndhB* gene, NAD) messenger ribonucleic acid (mRNA) by RT-PCR [31] were used to determine the presence and quality of the extracted RNA. Each sample was tested for the presence of three viroids and 34 viruses using specific RT-PCR and genus/family-based RT-PCR or nested RT-PCR/PCR tests as described in the original citation (Table S1). Primers for detection of the LChV1 HSP70-like gene (LC1-9135F 5’-TCTGCTGCTGCYATGCATCA-3’, LC1-9858R 5’-AWACACAAGCAGCAGTGGMA-3’) were designed for surveillance of little cherry disease during the outbreak in Tasmania in 2014 and were also used in this study. Cycling conditions for these primers were reverse-transcription at 48 °C for 30 min, then denaturation at 94 °C for 2 min, then 35 cycles of denaturation at 94 °C for 30 s, annealing at 55 °C for 30 s and extension at 72 °C for 45 s followed by a final extension at 72 °C for 10 min. Positive controls were included in every RT-PCR or PCR test and consisted of RNA extracted from lyophilised tissues of infected plants or synthetic positive controls designed using the GeneArt® Strings™ DNA Fragments (Invitrogen) and reverse-transcribed into RNA as previously described [32]. Negative controls and/or a water control were also used in every test.

The endpoint PCR products were electrophoresed in a 1% agarose gel using 0.5× TBE buffer and visualised using a UV transilluminator after staining with SYBR®Safe DNA Gel Stain (Invitrogen). Selected specific RT-PCR and all genus/family-based RT-PCR products were purified using the Qiaquick PCR purification kit (Qiagen, Chadstone, VIC, Australia), cloned using the pGEM-T Easy Vector system (Promega, Hawthorne, VIC, Australia) and sequenced using the ABI BigDye Terminator Version 3.1 kit on an AB3730xl sequencing machine (Applied Biosystems, Scoresby, VIC, Australia). The resulting sequences were analysed using BLASTn [33] to determine their identity.

### 2.3. Virus Metagenomic High-Throughput Sequencing (HTS) and Sequence Reads Analysis

A total of 24 *Prunus* trees were tested using virus metagenomic HTS and included 20/100 samples from the survey that were selected based on the *Prunus* host species, origin and virus species detected by RT-PCR during the survey, along with an additional four LChV2-infected cherry tree samples (LV16, LV27, LV35 and LVV) from Tasmania that had been submitted to the Tasmanian Plant Biosecurity and Diagnostics Branch to investigate the cause of little cherry symptoms. Five µL aliquots of TNA extract from each of the selected samples were used to prepare 24 individual metagenomic NGS libraries using NEBNext® Ultra™ RNA Library Prep Kit (New England BioLabs, Notting Hill, VIC, Australia) following the manufacturer’s instructions. The size distribution and concentration of the libraries were determined using the 2200 TapeStation® system (Agilent Technologies, Mulgrave, VIC, Australia) and Qubit® Fluorometer 2.0 (Invitrogen), respectively. The libraries were sequenced using the Illumina MiSeq with a paired read length of 2 × 251 bp. The generated raw sequence reads were quality trimmed, paired, assembled into contigs and BLASTn and BLASTx analysis of the assembled contigs was carried out as previously described [34]. Specific RT-PCR primers were designed to amplify overlapping regions of the virus genomes that were identified by HTS and confirm the predicted arrangement of the contigs after Sanger sequencing (Table S2). The RT-PCR products were directly sequenced or cloned using the pGEM-T Easy Vector system (Promega) and sequenced using the ABI BigDye Terminator Version 3.1 kit on an AB3730xl sequencing machine (Applied Biosystems) as previously described.

### 2.4. Phylogenetic and Sequence Identity Analysis

Full-length genome sequences of each virus species assembled from metagenomic HTS were aligned with each other, where multiple sequences were obtained, and with full genome sequences of all corresponding isolates available in GenBank (Table S3) using MUSCLE (version 3.8.31) [35]. Maximum likelihood phylogenetic trees were constructed in MEGA (version 6) [36] with 1000 bootstrap replicates and the resulting trees were visualised in FigTree (version 1.4.2) [37]. Branches having less than 50% bootstrap support were collapsed.

### 2.5. Recombination Analysis

The aligned full genome sequences of virus species from this study and the sequences of corresponding virus species isolates available in GenBank were checked for potential recombination events using RDP [38], GENECONV [39], BootSCan [40], MaxChi [41], Chimaera [42], SiSCan [43] and 3Seq [44] programs implemented in the RDP4 software [45]. Potential recombination events detected were considered to be statistically significant if detected by at least four programs with *p* values <0.05 [46].

## 3. Results

### 3.1. Virus and Viroid Detection by RT-PCR

Viruses and viroids were detected in 70% (70/100) of *Prunus* tree samples using species-specific or genus/family-based RT-PCR or nested RT-PCR/PCR tests (Table 2). The viruses and viroids that were already known to occur in Australian *Prunus* trees and which were also detected in samples from this survey included ACLSV, ApMV, APCLSV, ASGV, CVA, PDV, PNRSV, HSVd and PLMVd (Table 2). The viruses APLPV, ASPV and CMV were not detected. Viruses previously considered exotic to Australia, including CGRMV, CNRMV, LChV2 and PBNSPaV were detected for the first time in Australia (Table 2). Using a combination of species-specific and genus/family-based tests, PNRSV was most frequently detected (52/100 trees) compared to all other viruses and it was found in all *Prunus* species tested (almond, apricot, cherry, nectarine, peach and plum; Table 2).

The following viruses and viroids of almonds and/or stone fruit species, which are of quarantine concern to Australia, were not detected in Australian *Prunus* trees using species-specific or genus/family-based RT-PCR or nested RT-PCR/PCR tests during the survey: ApLV, ArMV, APV1, APV2, APV3, CLRV, CMLV, CRMaV, CTLaV, LChV1, PPV, PRMV, PcMV, RRSV, SLRSV, TBRV, TBSV, ToRSV and ASSVd (Table S4).

Some virus species required a combination of both species-specific and genus/family-based tests for detection to ensure all strains were detected (Table 2). For example, ACLSV was detected in 13 trees, but only 7/13 were detected with a specific test and 6/13 were detected with a genus/family-based test and none of the strains were detected with both tests. Similarly, 2/52 PNRSV isolates were only detected using the *Ilarvirus* test [47]. The two APCLSV isolates were only detected using the genus/family-based PDO RT-PCR test [20]. Conversely, some viruses including ApMV and PBNSPaV were only detected using species-specific tests. Sanger sequencing and BLASTn analysis of amplicons confirmed the detection of the viruses detected by the specific or genus/family-based RT-PCR tests. ACLSV and CVA isolates that were only detected by the genus/family-based RT-PCR tests in this study, shared lower percentage nucleotide identity of 80%–92% and 74%–97% respectively, with published GenBank isolate sequences (Table 2). The two APCLSV isolates from this study, which were only detected using the genus/family-based RT-PCR test had the highest nucleotide identity of less than 85% with GenBank isolate sequences (Table 2). A similar percentage of nucleotide identities were observed in genus/family-based RT-PCR amplicons (84%–98%) of PNRSV (Table 2).

Mixed infections of viruses and/or viroids were observed in 24/100 *Prunus* trees tested. Most of the mixed infections (17/24 trees) were observed in cherry trees (Table 3). PNRSV was most frequently found in mixed infection compared to other viruses and was detected in all the mixed infections. Other viruses that were also found frequently in mixed infections included CVA in 11/24 trees, and ACLSV and PBNSPaV in 4/24 trees each. PNRSV and CVA mixed infections occurred more frequently (6/24 trees) than other types of mixed virus infections.

### 3.2. HTS Analysis

After quality trimming and pairing of raw metagenomics HTS reads, 1,273,350–6,048,632 sequence reads from the 24 *Prunus* trees samples were used for downstream bioinformatic analysis (Table 4). The number of viral contigs generated from de novo assembly of the sequence reads from each of the 24 samples ranged from 1585–7973 contigs (Table 4). A BLASTn and BLSTx search of the GenBank database [33] revealed viral contigs in the 24 samples that covered full genomes of ACLSV (6/24), APCLSV (2/24), CVA (14/24), CGRMV (7/24), CNRMV (3/24), LChV2 (6/24) and PBNSPaV (7/24) (Table 4) and confirmed their detection by RT-PCR in the same samples.

Full genome contigs of AVCaV (2/24), ApLV (1/24), APV2 (1/24), LChV1 (5/24) and NSPaV (2/24), which were not detected in any of the 24 samples using species-specific RT-PCR or genus/family-based RT-PCR or nested RT-PCR/PCR, were also assembled (Table 4). HTS also identified additional CVA CGRMV, CNRMV, LChV2 and PBNSPaV positive *Prunus* trees that were not detected using species-specific or genus/family-based RT-PCR (Table 4).

There was evidence of multiple strains of virus species in individual samples: 4/24 samples had full genomes of two CVA strains, 1/24 samples had full genomes of three CVA strains and 1/24 samples had full genomes of two CGRMV strains and two LChV1 strains. (Table 4). The assembled full genome sequences of each virus and strains were further confirmed by RT-PCR using specific overlapping primers and Sanger sequencing. The confirmed full genome sequences of each virus were submitted to GenBank and the accession number of each virus RNA sequence is included as Supplementary Data (Table S5). The genomes of ApMV, PDV and PNRSV assembled from 15/24 *Prunus* tree samples in this study have been previously reported [34,48,49] and were not used for downstream analysis (Table 4).

### 3.3. Phylogenetic and Sequence Identity Analysis

Table 5 shows the range of nucleotide (nt) sequence identity between the genomes of Australian isolates and the similarity between Australia isolates and those published on GenBank. In most cases, the range of identities between genomes was smaller between Australian isolates compared to the range of identities between Australian and overseas isolates. Phylogenetic trees were also produced to show relationships between isolates (Figures S1–S5).

ACLSV: Three distinct clusters were observed in the phylogenetic analysis of genome sequences of the six Australian ACLSV isolates and genome sequences of 22 ACLSV isolates available from GenBank, which represented diverse hosts and geographic ranges (Figure S1). Five of the six Australian ACLSV isolates from this study (VIC10, LVV, TAS1, LV35 and LV27) were from cherry trees and occurred in one cluster, sharing 95%–97% nt identity with each other. They clustered with and shared 67%–95% nt identity with isolates from Asia and Europe. The Australian ACLSV peach isolate (WA1) shared 79% identity with the five ACLSV cherry isolates from this study and occurred in a separate phylogenetic group with a South Korean peach isolate (GenBank accession KY310578) with which it shared 94% nt identity (Figure S1; Table 5 and Table S6). All the Australian ACLSV isolates had the lowest nt identity range of 67%–68% with the highly divergent US ACLSV peach isolate (GenBank accession EU223295) which phylogenetically groups with APCLSV (Figure S1; Table 5 and Table S6).

APCLSV: The genomes of the two Australian APCLSV isolates from plum (VIC3) and nectarine (VIC11) obtained in this study had 96% nt identity and they clustered with a South Korean peach isolate (GenBank accession KY310579) and shared 96% nt identity and 98% nt identity, respectively, with this isolate (Figure S1; Table 5 and Table S6).

AVCaV: Two distinct clusters of AVCaV isolates were observed in the phylogenetic analysis of genome sequences from Australia and overseas (Figure S1). The Australian plum (QLD2) and nectarine (VIC3) AVCaV isolates shared 86% nt identity and occurred in separate clusters (Figure S1; Table 5 and Table S6). The Australian plum AVCaV isolate was most closely related (98% nt identity) to a French plum isolate (GenBank accession KM507062). The Australian AVCaV nectarine isolate most closely related (99% nt identity) to a previously reported Australian plum isolate (GenBank accession KY132099).

CGRMV: Eight CGRMV genomes were obtained by HTS from six Australian cherry samples and one peach sample. Two CGRMV strains, LV27_S1 and LV27_S2, were found in one cherry sample and shared 81% nt identity. All Australian isolates shared 80%–98% nt identity amongst themselves (Figure S2; Table 5 and Table S6) and 80%–96% with isolates from other countries (Table 5 and Table S6). The phylogenetic analysis revealed two distinct clusters of Australian CGRMV genomes, each containing three cherry isolates, and isolates within each cluster shared 97%–98% identity (TAS12, LV35 and LVV) or 91%–95% (TAS16, LV16 and LV27_S1). The two additional Australian CGRMV isolates (VIC5 and LV27_S2) occurred separately (Figure S2). LV27_S2 was the most divergent isolate and had the highest nt identity (82%) to the Australian cherry isolate LV16 (Figure S2; Table 5 and Table S6). The Australian CGRMV isolate from peach (VIC5) grouped with a USA cherry isolate (GenBank accession AF017780) and they shared 89% nt identity (Figure S2; Table 5 and Table S6).

CNRMV: The phylogenetic analysis revealed two distinct clusters of CNRMV isolates and all three Australian cherry isolates occurred in one group (Figure S2). Australian isolates LV35 and LVV had 99% nt identity and were most closely related (95% nt identity) to two Japanese CNRMV cherry isolates (Figure S2; Table 5 and Table S6). The Australian cherry CNRMV isolate, LV27, clustered with a South Korea peach isolate (GenBank accession KY310583) sharing 94% nt identity and 92% nt identity to other two Australian isolates.

ApLV: The phylogenetic analysis revealed two distinct clusters of ApLV isolates. The Australian ApLV apricot isolate from this study clustered with apricot isolates from Italy and France (Figure S2). The Australian apricot isolate had the highest (93%) nt identity to an Italian apricot isolate (GenBank accession HQ339956; Table 5 and Table S6).

APV2: The phylogenetic analysis revealed two distinct clusters of APV2 isolates. The Australian APV2 apricot isolate from this study, VIC18, clustered with two apricot isolates from Japan (GenBank accession KT893295) and the USA (GenBank accession KR998049) and had 96% and 94% nt identity to these two isolates respectively (Figure S2; Table 5 and Table S6).

PBNSPaV: The Australian PBNSPaV isolates from this study had an 82%–98% nt identity amongst themselves and a nt identity range of 71%–99% with GenBank isolates (Table 5 and Table S6). The phylogenetic analysis revealed two distinct clusters of PBNSPaV (Figure S3). Four Australian cherry and plum isolates (LV27, TAS12, VIC3 and QLD2) clustered in one group with two French plum isolates (GenBank accession KC590346 and KC590345) with a nt identity range of 95%–99% (Figure S3). The other three Australian PBNSPaV isolates (LVV, VIC2 and QLD13) were found in two cherry and one plum tree and shared 98% nt identity and were most closely related to a PBNSPaV plum isolate (GenBank accession EF546442) from the USA with a nt identity range of 98%–99%.

LChV1: The Australian LChV1 isolates had 76%–87% nt identity amongst themselves and 72%–99% nt identity with GenBank isolates (Table 5 and Table S6). LChV1 phylogenetic analysis revealed two distinct clusters and Australian isolates occurred in both clusters (Figure S3). Four Australian isolates (QLD13, TAS16, LV16 and LVV) occurred in a large cluster and the Australian isolates QLD13, TAS16 and LV16 formed a sub-cluster with Spanish cherry isolate (GenBank accession MH300061) and shared 88%–90% nt identity (Figure S3 and Table S6). Australian isolate LVV occurred in a separate sub-cluster with a Spanish cherry isolate (GenBank accession KX192366) and they had 99% nt identity. The Australian cherry isolate LV27 consisted of two strains (LV27_S1 and LV27_S2) which had 79% nt identity and occurred in different groups. LV27_S1 grouped with two cherry isolates from Italy (GenBank accession EU715989) and Germany (GenBank accession Y10237), sharing 93% and 94% nt identity respectively (Table 5 and Table S6). LV27_S2 grouped with and had 90% nt identity with a Japanese cherry isolate (GenBank accession MG934545) (Figure S3 and Table S6).

LChV2: The Australian LChV2 isolates from this study shared 76%–96% nt identity and phylogenetically clustered into two distinct clusters (Figure S3; Table 5 and Table S6). One group consisted of three Australian isolates (LV27, TAS12 and TAS16) and clustered with two cherry isolates from Germany (GenBank accession MF069043) and China (GenBank accession MG881767) sharing 83%–96% nt identity (Figure S3; Table 5 and Table S6). The second cluster consisted of three Australian isolates (LV27, TAS12 and TAS16) that were 95% identical and clustered with two cherry isolates from Belgium (GenBank accession MK803502 and MK895513) and one isolate from the USA (GenBank accession AF531505). Isolates within this cluster shared 89%–94% nt identity (Figure S3; Table 5 and Table S6).

NSPaV: The phylogenetic analysis revealed two distinct clusters of NSPaV and the Australian isolates are represented in both clusters (Figure S4; Table 5 and Table S6). The VIC5 isolate from peach was most closely related (96% nt identity) to a peach isolate from South Korea (GenBank accession MF326520) and the TAS17 isolate was most closely related (96% nt identity) to two nectarine isolates from the USA (GenBank accession KT273409 and KP638562).

CVA: The genomes of 20 Australian CVA isolates from apricot, cherry, peach and plum were obtained by HTS, from 13/14 trees that also tested positive by specific or genus/family-based RT-PCR. The 1/14 tree was not used for HTS. Phylogenetic analysis resulted in six distinct clusters of CVA isolates. CVA isolates from this study were distributed amongst all six clusters (Figure S5). CVA isolates from this study shared 80%–99% nt identity with each other and with GenBank isolates (Figure S5; Table 5 and Table S6). The Australian cherry tree samples LV35, TAS2, TAS4, TAS5 and TAS12 consisted of multiple full genome strains and each of the strains from the same tree occurred in different phylogenetic clusters (Figure S5). The cherry tree samples LV35, TAS2, TAS4, TAS5 each had two full CVA genomes and the two strains within each sample shared 80%–85% nt identity. TAS12 had three full CVA genomes which shared 80%–81% nt identity (Table 5 and Table S6).

### 3.4. Recombination Analysis

Recombination analysis of the full genome sequences of Australian isolates and GenBank isolate sequences identified recombination events in ACLSV, CGRMV, CVA, LChV1, LChV2 and PBNSPaV sequences (Table 6 and Table S7). Recombination events affected either coat protein (CP), heat shock (HSP), movement protein (MP) or replicase gene (Table 6 and Table S7). There were no recombination events identified in any of the Australian ApLV, APCLSV, APV2, AVCaV, CNRMV and NSPaV isolates.

All the recombination events detected in Australian ACLSV and CGRMV isolates, and in 4/6 LChV1, 6/7 LChV2 and 3/4 PBNSPaV Australian isolates had Australian isolates as their major parents while only 2/18 CVA recombination events had Australian major parents (Table 6 and Table S7). All the Australian recombinant isolates shared similar phylogenetic groupings with their parental isolates, whereas, in most cases the minor parental sequences occurred in separate phylogenetic groupings (Figures S1, S2, S3 and S6; Table 6 and Table S7).

## 4. Discussion

The number of *Prunus* trees tested in this study was not extensive, however, the approach of using a combination of virus species-specific and genus/family-based RT-PCR tests, RT-PCR, or nested RT-PCR/PCR and HTS allowed us to test Australian grown trees for many *Prunus* infecting viruses and to examine their genetic diversity. This study showed that viruses not previously reported in Australia were present in *Prunus* species, many of which should be excluded from programs that produce high-quality planting material for the Australian stone fruit and almond industries. This study highlighted the importance of understanding virus genetic diversity to ensure diagnostic tests can detect all known genetic strains of a virus species. It also highlighted the value of metagenomic HTS as a single diagnostic test that can indiscriminately detect all viruses in a single sample. Australia is a large continent and *Prunus* trees are grown in diverse regions throughout the country, with cool-temperate to Mediterranean climates, sometimes 1000’s of kilometres apart. Some virus species occur in many regions and sometimes closely related virus strains appear in different and distant regions and dissemination of the viruses by propagation material is probably the primary pathway. *Prunus* material is imported from many countries including Europe and the USA and is also a likely contributor to the diversity of species and genetic diversity within species observed in this study.

One or a combination of viruses were found in 70% of the trees that were tested. PNRSV, PDV, ApMV and ACLSV were detected in this study. These viruses have long been known to occur in many *Prunus* species in Australia and are included in certification testing for production of high health planting material, particularly in almonds [50,51]. Other endemic *Prunus* viruses and viroids including APCLSV, APLPV, ASGV, CVA, HSVd and PLMVd, were mainly found in the stone fruit and ornamental *Prunus* trees, but not in almonds. During the survey ApLV, APV2, CGRMV, CNRMV, LChV1, LChV2, NSPaV and PBNSPaV were detected in stone fruit species and this is the first report of these viruses in Australia. PNRSV was the most commonly detected virus, occurring in 52% of the samples tested. All other viruses that were detected occurred much less frequently (≤14%).

Except for LChV2, vectors are not known for most of the *Prunus* infecting viruses that were found in this study. The known mealybug vectors of LChV2 (*Phenacoccus aceris* and *Pseudococcus maritimus*) [52] are not known to occur in Australia. However, all viruses are transmitted vegetatively, during propagation and grafting, and some are transmitted in pollen, including PNRSV and PDV [53] and these are likely to be the primary means of distribution of *Prunus* infecting viruses in Australia. The high prevalence of PNRSV is likely to be due to a combination of the use of untested planting material, especially as the use of certified material is voluntary, and field transmission via pollen and thrips which occurs in Australia [54,55]. Although PDV is also pollen and vegetatively transmitted, similar to PNRSV, its low incidence (3%) in this study similar to other studies [34] may be due to its *Prunus* limited host range compared to PNRSV, which can also naturally infect other plant hosts [56,57,58].

ASPV and CMV were not detected although they are known to occur in other crops in Australia. This result was anticipated as they appear to be minor viruses of *Prunus* that are reported infrequently [59,60,61] and do not seem to be tested in overseas certification programs. The following exotic viruses and viroids were not detected: ASSVd, ArMV, APV1, APV3, CLRV, CMLV, CRLV, CRMV, CTLaV, HSVd, PLMVd, PcMV, PRMV, PPV, RpRSV, SLRSV, TRSV, TBRV, TBSV and ToRSV. As a result, imported germplasm is likely to continue to be tested for these viruses during Australian PEQ. However, it is possible the viruses that were not detected are present in Australia but at low prevalence and too few trees were tested in this study to allow detection.

Although ASGV is known to infect *Prunus* species in other countries [62], this study presents the first evidence that ASGV, which frequently infects Australian pome fruit species [63], can also infect *Prunus* species (plum) in Australia. Infection could have been through imported infected planting material, but it may also have occurred through mechanical transmission through the sharing of equipment between *Prunus* and other fruit crops such as citrus or pome fruit, where their growing regions overlap in Australia [64].

It is difficult to estimate when ApLV, APV2, CGRMV, CNRMV, LChV1, LChV2, NSPaV and PBNSPaV, which were initially considered exotic, were introduced into Australia, but it is possible they have been present for many years but gone undetected until this study. Indeed, the Victorian plum tree infected with PBNSPaV, ACLSV, ASGV and PNRSV was estimated to be more than 60 years old, which suggests that PBNSPaV may have been present for more than 60 years. These viruses were most likely introduced into Australia before they were known and included as part of the Australian import molecular testing regime. The associated symptoms were probably not observed by biological indexing during Australian PEQ because symptoms were not well described for biological indicator species or the indicators were symptomless. This is especially the case for recently described viruses such as NSPaV, that are only now being discovered through the development and implementation of HTS in association with diseases that previously had an unknown aetiology [24,65]. Therefore, as new pathogens and updates in pathogen biology and disease aetiology occur, it is important that there is continual surveillance of the literature to ensure these PEQ pathogen lists remain current to support the Australian *Prunus* industry’s biosecurity continuum.

The lack of detection of some viruses and viroids and low prevalence of others in Australia, especially those that are primarily spread through vegetative propagation, is likely to be a result of certification programs established in the 1970s [51,66] in combination with strict quarantine measures that adapt to include new virus species as they are reported. Testing is done during PEQ for many exotic viruses and exotic diseases with a suspected virus aetiology, using specific tests and/or biological indexing. In line with the remit of Australian quarantine, testing for non-regulated pests may not be done unless requested by importers or if a general test detects them. Detection of an endemic virus during PEQ may not preclude the entry of new germplasm but allows importers to decide how they would like to proceed with importation, directing it to a germplasm collection, undertaking virus eradication prior to distribution of material or discarding the material. New germplasm may enter certified pathogen-tested collections, which aim to ensure that the material distributed to industry is free of the significant viruses that occur in Australia like ACLSV, ApMV, PNRSV and PDV. The Australian almond industry tests almond trees and rootstocks annually for significant viruses, which minimises their spread and impact on production. However other Australian *Prunus* germplasm collections may only be tested for endemic viruses upon entry into their collection or tested infrequently, increasing the risk of distributing virus-infected material to nursery and production industries, especially for those viruses like PNRSV, which are also spread by pollen [67,68].

Like many other studies [69,70,71,72], mixed infections of two or more viruses were also detected in 24/100 *Prunus* trees tested. PNRSV occurred in all mixed infections which is not surprising given that PNRSV is easily transmitted by pollen and vegetatively, and was the most frequently detected virus [67,68]. Interestingly, no mixed infections were detected in almond trees which may be attributed to the rigorous certification testing regime undertaken by the Australian almond industry. In contrast, mixed infections were frequently detected in stone fruits with most mixed infections, sometimes as many as five viruses were detected in cherry trees e.g in Table 4 isolate TAS12, LV27, LV35 and LVV. Unlike the Australian almond industry, no formal certification virus testing program exists for other Australian *Prunus* industries and trees may not be tested or are tested infrequently instead of annually for viruses. Until this survey was completed in 2015, most Australian *Prunus* trees were tested only for ApMV, ACLSV, PDV and PNRSV because other viruses were not known to occur or were considered less significant. This infrequent testing, and only for a few viruses, especially before 2015, explains the high incidence of mixed infections observed in stone fruits in this study, especially in cherries, and highlights the need for these *Prunus* industries to undertake a more proactive virus testing program in which testing is undertaken for all viruses now known to occur in Australia.

In this study, the reliability of RT-PCR and PCR diagnostic tests was assessed to ensure they were fit for purpose for routine diagnostic testing of Australian *Prunus* trees for viruses. RT-PCR and PCR tests were chosen for this survey by comparing primers against available sequences in GenBank to determine their likelihood for detecting targeted virus, however, this process is only as good as the virus sequences available for comparison. Genetic similarity or variability of viruses and co-extracted nucleic acids may influence the reliability of RT-PCR and PCR tests resulting in false-positive or negative results. False-positive results can occur when the PCR primers have a high degree of similarity to other genetic material, including that of the host plant or other organisms within a geographic region [73]. For instance, there was a large discrepancy in the detection of the trichoviruses APCLSV and ACLSV using the specific and genus/family-based RT-PCR tests, where all APCLSV and 6/13 ACLSV detections were only by the genus/family-based PDO RT-PCR test [20]. Their amplicons had less than an 85% nucleotide identity with published APCLSV and ACLSV isolates, indicating that they were quite divergent. Similar variation in the detection of these trichoviruses has been previously reported and can be attributed to their high molecular variability which impacts the binding capacity of highly specific primers of the species-based RT-PCR tests, especially those used in this study [74,75,76]. Consequently, it is recommended that both virus species-specific and genus or family-specific RT-PCR tests are used to ensure detection of virus species in the genus *Ilarvirus, Foveavirus, Trichovirus, Capillovirus* and *Ampelovirus* in *Prunus* trees to overcome the risk of false-negative results, until improved tests can be developed. This study highlighted the need for further improvements in the development, selection and adoption of RT-PCR tests for detection of viruses in Australian grown *Prunus* species.

HTS was used to confirm the first detection of CGRMV, CNRMV, LChV2 and PBNSPaV in Australia by RT-PCR and PCR. It was also useful to confirm the presence of viruses detected by a genus- or family-based RT-PCR test but not by the specific test (e.g., APCLSV). Metagenomics HTS detection of additional virus species, specifically ApLV, AVCaV, APV2, NSPaV and LChV1, and strains of CGRMV, CNRMV, LChV2 and PBNSPaV, which were not detected by species-specific or genus- or family-based RT-PCR tests, highlights the high potential for a false-negative diagnosis by RT-PCR due to virus genetic variability. It also highlights the value of metagenomics HTS as a non-discriminative diagnostic tool for virus detection compared to RT-PCR, because it does not rely on knowledge of the virus genome sequences [77]. Given the high number of viruses that may need to be tested in some *Prunus* species, which could make RT-PCR a cost-prohibitive diagnostic tool, the reducing cost of HTS [28] makes this technology more attractive for routine virus detection in *Prunus* species in Australia.

In this study, full genomes of Australian ACLSV, APCLSV, AVCaV, ApLV, APV2, NSPaV, PBNSPaV, CNRMV, LChV2, LChV1, CGRMV and CVA isolates were generated by HTS, contributing to the global knowledge of their presence and diversity. Except for ApLV and APV2, multiple genome sequences were obtained for the other viruses. Multiple full-genomes of CVA, CGRMV and LChV1 strains were also detected by HTS in individual *Prunus* tree samples. Similar detection of multiple genetic strains or variants may also be achieved through traditional Sanger sequencing of cloned RT-PCR and PCR amplicons. However, limitations in the sequencing of a few clones due to time, labour and cost constraints may hamper the detections of some strains present in low titre mixed infections. This challenge in the detection of mixed virus genetic strains in a single plant has recently been overcome by HTS for other virus species [48,78,79], which further highlights the sensitivity and applicability of HTS in characterising and detecting virus genetic strains.

Phylogenetic and sequence identity analysis of full virus genomes was used to determine the genetic relationship between Australian *Prunus* virus isolates of ACLSV, APCLSV, AVCaV, ApLV, APV2, CGRMV, CNRMV, CVA, LChV1, LChV2, NSPaV and PBNSPaV from this study and isolates of each species previously published on GenBank. Where multiple strains of a species were identified, considerable genetic diversity was observed amongst the strains. As reported in other studies [18,72,76,80,81,82], these virus species were often distributed throughout their individual phylogenetic trees, rather than forming a single Australian cluster, and were related to overseas isolates originating from different regions. This suggests multiple introductions of each virus species into Australia. However, in some instances, for example, ACLSV, CVA, CGRMV, CNRMV, LChV1 and LChV2, clusters of some strains were observed, and this was most frequently associated with the cherry tree as a host. The original source of propagation material for each tree is not known, but it may be that where a cluster is observed in the same host, regardless of location, each of those trees was propagated from a single source tree and the genetic differences could be associated to evolution of the virus species with time. There is one exception, where two closely related LChV1 strains within a single cluster were from cherry and plum trees that were collected in Queensland and Victoria, respectively. This may be coincidental, or the two LChV1 strains may have a common origin. The common origin could be spread by a vector within an Australian *Prunus* germplasm collection or an overseas germplasm, although no vector is known for LChV1. It could also be associated with the use of a common infected rootstock.

The multiple genetic strains of CVA, CGRMV or LChV1 within individual trees were highly divergent from each other and occurred in different phylogenetic clusters. For example, the three distinct CVA strains identified in sample TAS12 occurred in the three of the six major CVA clusters reported by [83]. Co-infection of divergent virus strains is known [84] and is likely to represent multiple infection events, which could be through grafting or transmission by an insect vector, although no vector is known for CVA, CGRMV and LChV1. The biological implication of infection with multiple distinct virus genetic strains is not known and further studies are required.

Recombination is a powerful evolutionary driving force that results in the generation of new genotypes in RNA viruses [85,86]. Recombination events have been previously reported in RNA viruses in the family *Betaflexiviridae* and *Closteroviridae* [79,84,87,88] and in this study, multiple recombination events were identified amongst Australian isolates of ACLSV, CGRMV, CVA, LChV1, LChV2 and PBNSPaV. All the Australian recombinant isolates shared similar phylogenetic groupings with their major parental sequence which indicates close genetic connectivity within the population of these recombinants. Previous studies have shown frequent recombination events between multiple divergent virus genetic strains occurring in individual plants [79,81]. Interestingly, no recombinants were observed in the six *Prunus* trees that had multiple full-genome genetic strains of CVA, CGRMV and LChV1. This could indicate recent introductions of these viruses into Australia or recent multiple or single infection events.

## 5. Conclusions

The comprehensive survey of incidence and genetic diversity of *Prunus* viruses in Australia conducted by this study provides useful information to support Australian *Prunus* industry biosecurity. This study has identified 18 viruses and viroids occurring in Australia *Prunus* species and provides the first report of ApLV, APV2, CGRMV, CNRNV, LChV1, LChV2, NSPaV and PBNSPaV in Australia. This information has been used to update the quarantine status of viruses and viroids in Australian *Prunus* trees. The low prevalence of some viruses in Australia suggests that quarantine and certification have been useful in limiting their distribution, particularly in almonds and stone fruit species other than cherry. However, the use of certified material is voluntary and the use of untested planting material in combination with field transmission via pollen would explain the higher prevalence of PNRSV and highlights the importance of using pathogen tested material. Since the conclusion of the survey using RT-PCR in 2015, several new viruses of *Prunus* were reported including *Caucasus prunus virus* (CPrV), *Cherry virus F* (CVF), *Peach leaf pitting-associated virus* (PLPaV), *Prunus yellow spot-associated virus* (PYSaV), *Prunus virus T* (PrVT) and *Prunus virus F* (PrVF) [26,27,89,90,91]. Molecular tests should be developed and validated in Australia for detection of these viruses and surveillance should be done to determine their presence within Australian almond and stone fruit production areas. HTS is increasingly being applied for plant pathogen detection to support plant biosecurity and certification programs [77,92,93,94] and the results of this study further highlight its value as a non-discriminatory diagnostic alternative to traditional virus diagnostic methods such as PCR to support the Australian *Prunus* industry biosecurity.

## Figures and Tables

**Table 1:** The region within Australia in which samples were collected and the number of samples of each *Prunus species* that were tested for three viroids and 34 viruses.

Region	*Prunus* Species	No. of Samples
New South Wales	Almond (*P. dulcis*)	33
	Peach (*P. persica*)	1
Queensland	Apricot (*P. armeniaca*)	2
	Nectarine (*P. persica var. nucipersica*)	2
	Plum (*P. domestica*)	8
	Peach (*P. persica*)	3
	Sweet Cherry (*P. avium*)	1
South Australia	Nectarine (*P. persica* var. *nucipersica*)	3
	Peach (*P. persica*)	2
Tasmania	Apricot (*P. armeniaca*)	2
	Almond (*P. dulcis*)	1
	Peach (*P. persica*)	1
	Plum (*P. domestica*)	3
	Sweet Cherry (*P. avium*)	10
Victoria	Almond (*P. dulcis*)	1
	Apricot (*P. armeniaca*)	2
	Nectarine (*P. persica var. nucipersica*)	2
	Plum (*P. domestica*)	9
	Purple leaf plum (*P. cerasifera*)	1
	Peach (*P. persica*)	8
	Sweet Cherry (*P. avium*)	2
Western Australia	Apricot (*P. armeniaca*)	1
	Nectarine (*P. persica var. nucipersica*)	1
	Peach (*P. persica*)	1

**Table 2:** The virus and viroid species that were detected by specific and/or genus/family-based RT-PCR or nested RT-PCR/PCR in Australian *Prunus* trees, the frequency of detection by both tests, the highest percentage (%) BLAST nucleotide identity of sequenced amplicons from both tests and the hosts in which they were detected are shown.

Virus Species	Genus	Number Positive by Specific RT-PCR	Highest % Nucleotide Identity of the Specific RT-PCR Amplicon (s)	Number Positive by Genus/Family-Based RT-PCR or Nested RT-PCR/PCR	Highest % Nucleotide Identity of the Genus/Family RT-PCR Amplicon (s)	Number Positive Using Both Species and Genus/Family-Based RT-PCR or Nested RT-PCR/PCR	Total Positives	*Prunus* Host *
*Apricot pseudo-chlorotic leaf spot virus* (APCLSV)	*Trichovirus*	0/100	N/A	2/100	82–85%	0/100	2/100	Nectarine (1), Plum (1)
*Apple stem grooving virus* (ASGV)	*Capillovirus*	1/100	99%	1/100	98%	1/100	1/100	Plum (1)
*Apple chlorotic leaf spot virus* (ACLSV)	*Trichovirus*	7/100	Not sequenced	6/100	80–92%	0/100	13/100	Apricot (2), Cherry (3), Nectarine (1), Peach (2), Plum (5)
*Apple mosaic virus* (ApMV)	*Ilarvirus*	1/100	Not sequenced	0/100	N/A	0/100	1/100	Almond (1)
*Cherry green ring mottle virus* (CGRMV)	*Robigovirus*	4/100	96–99%	N/A	N/A	N/A	4/100	Cherry (3), Peach (1)
*Cherry necrotic rusty mottle virus* (CNRMV)	*Robigovirus*	2/100	96–97%	N/A	N/A	N/A	2/100	Cherry (2)
*Cherry virus A* (CVA)	*Capillovirus*	14/100	Not sequenced	10/100	74–97%	10/100	14/100	Apricot (4), Cherry (9), Plum (1)
*Little cherry virus 2* (LChV2)	*Ampelovirus*	3/100	97–98%	2/100	94–98%	2/100	3/100	Cherry (3)
*Plum bark necrosis stem pitting associated virus* (PBNSPaV)	*Ampelovirus*	4/100	95–99%	0/100	N/A	0/100	4/100	Cherry (1), Plum (3)
*Prune dwarf virus* (PDV)	*Ilarvirus*	3/100	Not sequenced	3/100	98%	3/100	3/100	Cherry (2), Peach (1)
*Prunus necrotic ringspot virus* (PNRSV)	*Ilarvirus*	50/100	Not sequenced	52/100	84–98%	50/100	52/100	Almond (19), Apricot (6), Cherry (9), Nectarine (4), Peach (3), Plum (11)
*Hop stunt viroid* (HSVd)	*Hostuviroid*	9/100	Not sequenced	N/A	N/A	N/A	9/100	Almond (1), Apricot (3), Nectarine (1), Plum (4)
*Peach latent mosaic viroid* (PLMVd)	*Pelamoviroid*	6/100	Not sequenced	N/A	N/A	N/A	6/100	Nectarine (4), Peach (2)

* Numbers in brackets are the number of trees in which the virus was detected.

**Table 3:** The number of each of the *Prunus* trees in which mixed infections of viruses were detected by RT-PCR tests, the total number of samples and detections of each combination of viruses are shown.

Virus Mixed Infections	Almond	Apricot	Cherry	Nectarine	Peach	Plum	Total Detection
PNRSV, ACLSV	-	-	3	-	-	-	3
PNRSV, CVA	-	-	6	-	-	-	6
PNRSV, PBNSPaV	-	-	1	-	-	2	3
PNRSV, ACLSV, CVA	-	-	2	-	-	-	2
PNRSV, ApMV, PBNSPaV, HSVd	-	-	-	-	-	2	2
PNRSV, CVA, HSVd	-	1	-	-	-	-	1
PNRSV, PDV, CVA	-	-	1	-	-	-	1
PNRSV, ACLSV, HSVd	-	-	-	-	1	-	1
PNRSV, LChV2, CGRMV	-	-	1	-	-	-	1
PNRSV, ACLSV, ASGV, PBNSPaV	-	-	-	-	-	1	1
PNRSV, CVA, CNRMV, CGRMV	-	-	1	-	-	-	1
PNRSV, CGRMV, LChV2, CVA, PBNSPaV	-	-	1	-	-	-	1
PNRSV, ACLSV, LChV2, CVA, CNRMV, CGRMV	-	-	1	-	-	-	1
**Total No. of samples**	**0**	**1**	**17**	**0**	**1**	**5**	**24**

**Table 4:** High-throughput sequencing (HTS) data from 24 *Prunus* tree samples including the host species and origin, number of reads, the number of viral contigs and the full genome viruses identified from BLASTn analysis are shown.

Isolate	*Prunus* Host	Origin	Virus Detected by PCR	No. of Reads	No. of Contigs	Full Genome of Each Virus Assembled
QLD 2 ^#^	Plum	Queensland	PNRSV, PBNSPaV	1,676,855	3767	AVCaV, PBNSPaV, PNRSV ^#^
QLD 11 ^#^	Cherry	Queensland	PNRSV, PDV, CVA	1,644,093	3958	CVA, PDV ^#^, PNRSV ^#^
QLD 13 ^#^	Plum	Queensland	PNRSV, PBNSPaV	2,197,080	3279	LChV1, PBNSPaV, PNRSV ^#^
TAS 1 ^#^	Cherry	Tasmania	PNRSV, ACLSV, CVA	1,273,350	2833	ACLSV, CVA, PNRSV ^#^
TAS 2 ^#^	Cherry	Tasmania	PNRSV, CVA	1,985,665	4445	CVA (2) *, PNRSV ^#^
TAS 4 ^#^	Cherry	Tasmania	PNRSV, CVA	1,528,988	2314	CVA (2) *, PNRSV ^#^
TAS 5 ^#^	Cherry	Tasmania	PNRSV, CVA	1,687,727	3017	CVA (2) *, PNRSV ^#^
TAS 10 ^#^	Apricot	Tasmania	PNRSV	1,471,799	3949	APV2, PNRSV ^#^
TAS 12 ^#^	Cherry	Tasmania	PNRSV, CGRMV, CVA,	2,709,497	2782	CGRMV, CVA (3) *, LChV2, PBNSPaV, PNRSV ^#^
TAS 16 ^#^	Cherry	Tasmania	PNRSV, CGRMV, LChV2,	1,982,006	1972	CGRMV, LChV1, LChV2, PNRSV ^#^
TAS 17 ^#^	Peach	Tasmania	PNRSV	2,461,022	2295	NSPaV, PNRSV ^#^
LV16	Cherry	Tasmania	CGRMV, CVA, LChV2	1,917,626	2437	CGRMV, CVA, LChV1, LChV2
LV27	Cherry	Tasmania	ACLSV, CGRMV, CVA, LChV2	6,024,907	7973	ACLSV, CGRMV (2) *, CNRMV, CVA, LChV1 (2) *, LChV2, PBNSPaV
LV35	Cherry	Tasmania	ACLSV, CGRMV, CNRMV, CVA, LChV2	6,048,632	6669	ACLSV, CGRMV, CNRMV, CVA (2) *, LChV2
LVV ^#^	Cherry	Tasmania	PNRSV, CGRMV, LChV2, CVA, PBNSPaV	5,875,197	7656	ACLSV, CGRMV, CNRMV, CVA, LChV1, LChV2, PBNSPaV, PNRSV ^#^
VIC 2 ^#^	Plum	Victoria	ApMV, PNRSV, PBNSPaV	1,499,807	3162	PBNSPaV, ApMV ^#^, PNRSV ^#^
VIC 3	Plum	Victoria	APCLSV	1,779,895	2710	APCLSV, AVCaV, PBNSPaV
VIC 5	Peach	Victoria	CGRMV	1,643,124	1585	CGRMV, NSPaV
VIC 10 ^#^	Cherry	Victoria	PNRSV, ACLSV	1,648,521	4724	ACLSV, CVA, PNRSV ^#^
VIC 11	Nectarine	Victoria	APCLSV	2,048,918	4547	APCLSV
VIC 12	Plum	Victoria	CVA	2,534,523	5922	CVA
VIC 18	Apricot	Victoria	CVA	2,492,417	5972	ApLV, CVA
WA 1 ^#^	Peach	Western Australia	PNRSV, ACLSV	2,416,636	5958	ACLSV, PNRSV ^#^
WA 2	Apricot	Western Australia	CVA	1,596,837	5452	CVA

* The number in brackets indicates the number of full genomes of virus strains assembled. ^#^ *Prunus* tree samples used in other published HTS studies on ApMV, PDV and PNRSV and were not used for downstream analysis [34,48,49].

**Table 5:** The percentage (%) nucleotide identity comparison of full genome sequences of isolates detected in this study by metagenomic HTS and corresponding full genome sequences of isolates available in GenBank are shown.

Family	Virus Species	No. of Australian Isolates	% Nucleotide Identity Range between Australian Isolates	% Nucleotide Identity Range between Australian and GenBank Isolates
*Trinivirinae*	ACLSV	6	79%–97%	67%–95%
	APCLSV	2	96%	81%–98%
	AVCaV	2	86%	86%–99%
	CVA	20	80%–99%	80%–99%
*Quinvirinae*	ApLV	1	-	75%–93%
	APV2	1	-	79%–96%
	CNRMV	3	92%–99%	86%–95%
	CGRMV	8	80%–98%	80%–96%
*Closteroviridae*	PBNSPaV	7	82%–98%	71%–99%
	LChV2	6	76%–96%	77%–96%
	LChV1	6	76%–87%	72%–99%
*Luteoviridae*	NSPaV	2	94%	94%–96%

**Table 6:** The number of recombinants and recombination events identified using the RDP4 package within full genome sequences of Australian *Apple chlorotic leaf spot virus* (ACLSV), *Cherry green ring mottle virus* (CGRMV), *Cherry virus A* (CVA), *Little cherry virus 1* (LChV1), *Little cherry virus 2* (LChV2) and *Plum bark necrosis stem pitting-associated virus* (PBNSPaV) isolates are shown.

Virus Species	No. of Isolates	No. of Recombinants	No. of Recombination Events	Genes Affected
ACLSV	6	5	10	MP, RdRp
CGRMV	8	3	8	CP, Hel, RdRp
CVA	20	6	17	CP, MP, RdRp
LChV1	6	3	5	CP, HSP70
LChV2	6	5	7	Replicase
PBNSPaV	7	3	4	CP, RdRp

## Supplementary Materials

Supplementary materials can be found at https://www.mdpi.com/1999-4915/12/2/246/s1.
