# Supplementary material for: Updating the Quarantine Status of Prunus Infecting Viruses in Australia
Source: Viruses. 2020 Feb 23;12(2):246. doi: 10.3390/v12020246 (PMC7077234; doi:10.3390/v12020246)
Supplement: Supplementary file 1 [file viruses-12-00246-s001.pdf]

Supplementary data

Table S1. The list of *Prunus* infecting viruses and viroids that were tested in each sample using specific RT-PCR and genus/family-based RT-PCR or nested RT-PCR/PCR, the expected product size and the reference for each test.

**Endemic viruses**	**Genus**	**Expected product size**	**Reference**
American plum line pattern virus (APLPV)*	*Ilarvirus*	563bp	Sanchez-Navarro, *et al.* [98]
Apple chlorotic leaf spot virus (ACLSV)	*Trichovirus*	358bp	Candresse, Lanneau, Revers, Macquaire, German, Dunez, Grasseau and Malinovsky [78]
Apple mosaic virus (ApMV)	*Ilarvirus*	261bp	Petrzik and Svoboda [99]
Apple stem grooving virus (ASGV)	*Capillovirus*	456bp	Ito, *et al.* [100]
Apple stem pitting associated virus (ASPV)	*Foveavirus*	370bp	Menzel, *et al.* [101]
Apricot latent virus (ApLV)*	*Foveavirus*	1500bp	García-Ibarra, *et al.* [102]
Apricot pseudo-chlorotic leaf spot virus (APCLSV)	*Trichovirus*	446bp, 631bp	Foissac, Svanella-Dumas, Dulucq, Candresse and Gentit [20],Liberti, Ragozzino, Gentit, Marais, Svanella-Dumas and Candresse [79]
Asian Prunus virus 2 (APV2)*	*Foveavirus*	262bp	Marais, Svanella-Dumas, Foissac, Gentit and Candresse [19]
Cherry green ring mottle virus (CGRMV)*	*Robigovirus*	958bp	Li and Mock [103]
Cherry necrotic rusty mottle virus (CNRMV)*	*Robigovirus*	584bp	Osman, Al Rwahnih, Golino, Pitman, Cordero, Preece and Rowhani [77]
Cherry virus A (CVA)	*Capillovirus*	566bp	Osman, Al Rwahnih, Golino, Pitman, Cordero, Preece and Rowhani [77]
Cucumber mosaic virus (CMV)	*Cucumovirus*	501bp	Wylie, *et al.* [104]
Little cherry virus 1 (LCHV1)*	*Velarivirus*	723bp	This study
Little cherry virus 2 (LChV2)*	*Ampelovirus*	438bp; 180bp	Rott and Jelkmann [105],Jelkmann, *et al.* [106]
Plum bark necrosis stem pitting associated virus (PBNSPaV)*	*Ampelovirus*	290bp; 400bp	Al Rwahnih, Uyemoto, Falk and Rowhani [83],Ghanem-Sabanadzovic, *et al.* [107]
Prune dwarf virus (PDV)	*Ilarvirus*	172bp	Parakh, *et al.* [108]
Prunus necrotic ringspot virus (PNRSV)	*Ilarvirus*	455bp	MacKenzie, McLean, Mukerji and Green [29]
**Exotic viruses and viroids species**	**Genus**	**Expected product size**	**Reference**

Apple scar skin viroid (ASSV)	*Apscaviroid*	267bp	Faggioli and Ragozzino [109]
Arabis mosaic virus (ArMV)	*Nepovirus*	290bp	Wetzel, *et al.* [110]
Asian Prunus virus 1 (APV1)	*Foveavirus*	262bp	Marais, Svanella-Dumas, Foissac, Gentit and Candresse [19]
Asian Prunus virus 3 (APV3)	*Foveavirus*	262bp	Marais, Svanella-Dumas, Foissac, Gentit and Candresse [19]
Cherry leaf roll virus (CLRV)	*Nepovirus*	416bp	Werner, *et al.* [111]
Cherry mottle leaf virus (CMLV)	*Trichovirus*	848bp	James and Upton [112]
Cherry rasp leaf virus (CRLV)	*Cheravirus*	429bp	James, *et al.* [113]
Cherry rusty mottle virus (CRMV)	*Robigovirus*	695bp	Villamor and Eastwell [23]
Cherry twisted leaf associated virus (CTLaV)	*Robigovirus*	562bp	Villamor and Eastwell [23]
Hop stunt viroid (HSVd)	*Hostuviroid*	450bp	Domingo, *et al.* [114]
Peach latent mosaic viroid (PLMVd)	*Pelamoviroid*	337bp; 340bp	Shamloul, *et al.* [115],Ambrós, *et al.* [116]
Peach mosaic virus (PcMV)	*Trichovirus*	383bp	James, *et al.* [117]
Peach rosette mosaic virus (PRMV)	*Nepovirus*	388bp	Lebas and Ward [118]
Plum pox virus (PPV)	*Potyvirus*	243bp	Wetzel, *et al.* [119]
Raspberry ringspot virus (RpRSV)	*Nepovirus*	800bp	Lebas and Ward [118]
Strawberry latent ringspot virus (SLRSV)	*Unassigned*	293bp	Faggioli and Ragozzino [109]
Tobacco ringspot virus (TRSV)	*Nepovirus*	320bp	Fuchs, *et al.* [120]
Tomato black ring virus (TBRV)	*Nepovirus*	73bp	Harper, *et al.* [121]
Tomato bushy stunt virus (TBSV)	*Tombusvirus*	1220bp	Russo, *et al.* [122]
Tomato ringspot virus (ToRSV)	*Nepovirus*	450 bp	Griesbach [123]
**Generic RT-PCR or nested RT-PCR/PCR tests**	**Genus**	**Expected product size**	**Reference**
Ampelovirus	*Ampelovirus*	580-620bp; 490bp	Maliogka, Dovas and Katis [47],Dovas and Katis [124]
Capillovirus	*Capillovirus*	446bp	Foissac, Svanella-Dumas, Dulucq, Candresse and Gentit [20]
Closteroviridae	*Closteroviridae*	580-620bp	Dovas and Katis [125]
Foveavirus	*Foveavirus*	363bp, 198bp	Dovas and Katis [124,125]
Foveavirus	*Foveavirus*	631bp	Foissac, Svanella-Dumas, Dulucq, Candresse and Gentit [20]
Ilarvirus	*Ilarvirus*	381bp	Maliogka, Dovas and Katis [47]
Trichovirus	*Trichovirus*	362bp	Foissac, Svanella-Dumas, Dulucq, Candresse and Gentit [20]

*Virus species that were considered exotic at the time of the survey but are now considered present in Australia.

Table S2. Specific overlapping primers used for RT-PCR amplification to confirm metagenomic high-throughput sequencing (HTS) generated genomes of *Apple chlorotic leafspot virus* (ACLSV), *Apricot latent virus* (ApLV), *Apricot pseudo-chlorotic leaf spot virus* (APCLSV), *Apricot vein clearing-associated virus* (AVCaV), *Asian prunus virus 2* (APV2), *Cherry virus A* (CVA), *Cherry green ring mottle virus* (CGRMV), *Cherry necrotic rusty mottle virus* (CNRMV), *Little cherry virus 1* (LChV1), *Little cherry virus 2* (LChV2), *Nectarine stem pitting-associated virus* (NSPaV) and *Plum bark necrosis stem pitting-associated virus* (PBNSPaV)

Apple chlorotic leaf spot virus (ACLSV)			
**Primer Name**	**Sequence**	**Tm °C**	**Product size base pair (bp)**
ACLSV_1F	GTGAGTAAACAGATTGACGTAACGC	62	790
ACLSV_1R	CCATTTTCCAGTGGCTGAGTGT		
ACLSV_2F	GTTTTGCCTTTCCTTTATCAGTTCGA	59	795
ACLSV_2R	GATCCACTTCTCTTCCAGGGGT		
ACLSV_3F	ACACCCCGAATGTCATTTTCTATGG	61	884
ACLSV_3R	CCTGTTTGATCTCCCCTGTTGC		
ACLSV_4F	GCTCAACTCAAGAATAGAAAAGCAGC	62	799
ACLSV_4R	GGTTCGACAAGCTTGAGCCTTT		
ACLSV_5F	GAAGATCATCAATGGATTCTCTTGCCA	57	871
ACLSV_5R	CCATAAAGTGGTGAGCAGATGGC		
ACLSV_6F	TTGCAAAGAAACTGGCCATAGAAAC	62	877
ACLSV_6R	CTTGCCTCTACTTTCTGCTGCC		
ACLSV_7F	TGGATGATGATTTAACTTTTCTTGCTGC	61	869
ACLSV_7R	CGCAAAACATCGGCACTTTTGT		
ACLSV_8F	GGATGATATGTGTGCCTTGAGGAA	57	795
ACLSV_8R	GAAGTCACCAATGTAGCCGAGC		
ACLSV_9F	TTTCGGGGTGGTGCTATGTGTA	56	864
ACLSV_9R	CTATGTTCGCGAAGATGGCCTC		
ACLSV_10F	TTCTCAGGAACAAAGGAAAAGGCA	62	790
ACLSV_10R	TGACTCTTTATACTCTTTCATGGGTTCA		
Apricot latent virus (ApLV)			
**Primer Name**	**Sequence**	**Tm °C**	**Product size base pair (bp)**
ALV_1F	CCGGGATACGCAAACAAACTCT	58	962
ALV_1R	CATAGCCACAGCCTCAAAACCA		
ALV_2F	TCTTATCAAAAGTAGTTTCTCCCATCACT	59	794
ALV_2R	AAGCAAGCACGTAGGTTAGCAG		
ALV_3F	GCCAAGATTGTTGTCTCGCTCT	62	809
ALV_3R	ATCATCTCGATGCATTGCCAGC		
ALV_4F	TCGCTTCCTTGATGATTTCTTGGA	62	910
ALV_4R	CCCCTTGCGTCACTTTTGGTTT		
ALV_5F	AAGGTTCATTCTCTGGCTTTCGA	61	824
ALV_5R	TTGATACCCGGTTCAGGTCTGA		
ALV_6F	AGAATCCCTGCTGAATTTTCTTTGGA	62	857
ALV_6R	CTTCCGCCCCATCATTGAGAAC		

ALV_7F	GTTTTTGTTGGGAGATTACCATGCA	56	845
ALV_7R	TTCAGCAGCTGGAGAGGAGAAG		
ALV_8F	GGTATGAAGCCATTTACCCAAGACA	58	881
ALV_8R	CAGTGAACTGGACTTTGGCCTT		
ALV_9F	TGCTGGTGATGATATGTGTGCA	61	834
ALV_9R	CCCAAATTCCGTAACACCTGCA		
ALV_10F	TGATCCCTACCAAAATCACGGTG	58	812
ALV_10R	GCTTAAGGCCCTTCACCAATTCT		
ALV_11F	AGAGTGAATTCATGCACCATTGTGA	61	802
ALV_11R	CAAGCGTGAAGGGGGTAATCAC		
ALV_12F	CCACTCCTCAGAGGGTGTTTCA	56	835
ALV_12R	ATAAAACACACTCTGCCGTCCC		
Apple pseudo-chlorotic leaf spot virus **(APCLSV)**			
**Primer Name**	**Sequence**	**Tm °C**	**Product size base pair (bp)**
APCLSV_1F	ACCAAATCAAATCCAAATCAACCGT	60	896
APCLSV_1R	ACAAACAAAGTGCCCACATCGA		
APCLSV_2F	ACTTGGCTCTCATCATTTGTTCCA	62	860
APCLSV_2R	ACGCACCTGGACCTTTCTTTTG		
APCLSV_3F	TGGAAGGAAAATTCTGAGGGTAGGA	57	911
APCLSV_3R	TCACTCTTCACCCCATGCTTGA		
APCLSV_4F	TGCGATTTTCTCAGTAAGTTGTCATGA	56	886
APCLSV_4R	CTACCAAAATGCCCCTTGACCC		
APCLSV_5F	CTACGGAGTCTTTGGGTTTGCA	59	857
APCLSV_5R	AGTTTGAGTGGGACCGATGACA		
APCLSV_6F	TTGAGAACATTGAAAGGTGGCCT	57	827
APCLSV_6R	TCTGCACCATGGGCTGAATTTC		
APCLSV_7F	TCCCAGCTGTGTACGAAATTTGA	56	870
APCLSV_7R	CATCGCCATCCTCGTCAGAAAG		
APCLSV_8F	CAGGTGCTGACAAGGTTCTTCA	61	804
APCLSV_8R	TTCTCTTGTGTGGAGGGCAGAT		
APCLSV_9F	TGTGGAGAACCTAGGACTTGACG	59	836
APCLSV_9R	GCTCAGGATGCTTACCACCAAC		
APCLSV_10F	GCCAGTGAGGATGAATTAGATGCT	61	795
APCLSV_10R	ACTCCTTTTATTGACTGCGACTCT		
Asian prunus virus 2 **(APV2)**			
**Primer Name**	**Sequence**	**Tm °C**	**Product size base pair (bp)**
APV2_1F	TATCAACAAACGACCCAGCTCG	60	871
APV2_1R	GCTTACAACATGGTGCGCAAAG		
APV2_2F	ACCCCTCAGCTCTGGTTACATC	62	811
APV2_2R	CAACGGATGCCACATAACTGCT		
APV2_3F	GCCTGAAGTTTTCAAGAGGCTG	62	820
APV2_3R	AGCACCTCGTGAAAAAGAAGCA		

APV2_4F	CGCCTCTTTGCCAACATTGTAA	57	932
APV2_4R	TGGCAAAGCAGGTTCAAAGTGT		
APV2_5F	TGCTTCCAGGCAATAATTTCTCTTATGA	59	839
APV2_5R	CCTCATCGAGAACCACACTTGC		
APV2_6F	CTCAATTCAAAGAGGGGGAGATCC	62	800
APV2_6R	ACCAGCAAGCTTGTCTTCCATG		
APV2_7F	ATTGTCAAACAACTCCCTGGCC	61	882
APV2_7R	CTGATTCAGTGCACATGCCAGT		
APV2_8F	GTCCTGCCAGCCAACTTGTATA	61	859
APV2_8R	GCTCCGGCAATACAGTGAACAA		
APV2_9F	TCTTGTGTTTGGTGATGGATTTTGTT	56	846
APV2_9R	CGTCCAAATAACTCCCACCGTG		
APV2_10F	GGGTGGGGGTTGTTATTTGGTG	57	828
APV2_10R	TCCACTTGTCGTTGCCTCAATG		
APV2_11F	CTCACAGCTTGATGAGGACCTT	62	791
APV2_11R	GTTCTGTGCAATGAAACCCCCT		
APV2_12F	CAAGTCTCAAACAATGCTGCGC	59	828
APV2_12R	GCCTGTCTCAAACTCCCAAAACT		
Apricot vein clearing-associated virus **(AVCaV)**			
**Primer Name**	**Sequence**	**Tm °C**	**Product size base pair (bp)**
QLD2AVCaV_1F	ACTTGAACTTCTCAATAGACTCCAACC	60	1055
QLD2AVCaV_1R	CTTGAGCTTAGCCATAGCGCTC		
QLD2AVCaV_2F	GGACTTTGTCCCAATAAGCAAAGATC	57	972
QLD2AVCaV_2R	TCTGCAAACATGCCTCCTGAAG		
QLD2AVCaV_3F	GGGGACACAATTCTGTTGAGTGG	57	890
QLD2AVCaV_3R	CTCCCAGTCATCAGCCAGGTTA		
QLD2AVCaV_4F	GGCAAAGGGAATGGAAAGGGTC	60	910
QLD2AVCaV_4R	TCCCTGTCAACCTCGTCCTTAC		
QLD2AVCaV_5F	GCTGTGATAAACAAGGGCCTTG	57	908
QLD2AVCaV_5R	CAGATCATCACCAGCGTGCTTT		
QLD2AVCaV_6F	TAAGAGCTCAGTTGCCTGGTGA	60	976
QLD2AVCaV_6R	ATGCAGACTTTGGCTTTGCAGT		
QLD2AVCaV_7F	TGGTGGGAATTTGGATGGTTCAC	60	958
QLD2AVCaV_7R	CTCTGCGACATTCCCAGCAATT		
QLD2AVCaV_8F	GAAGGAGCGGCAAGGAATTTGA	60	972
QLD2AVCaV_8R	GACCTTGCCAGTTTTGGTCAGT		
QLD2AVCaV_9F	GAGGGATTGGGTGAATGCACTG	56	900
QLD2AVCaV_9R	ACCCTCTACAATACTGTGGCCA		
VIC3AVCaV_1F	ACTTCTCAATAGACTCCAACCTTGAG	57	1069
VIC3AVCaV_1R	AGATCGTCACCCATAAGCTGCT		
VIC3AVCaV_2F	AGCAAAGATCTTGTGGAAAGAGTGT	62	958
VIC3AVCaV_2R	GATATGGCGATGCATGCTGGAA		

VIC3AVCaV_3F	GGAACTCTCTTTCCTTCAATTTGAGC	62	937
VIC3AVCaV_3R	CTGTGAGAATTGGGTAGCCAGC		
VIC3AVCaV_4F	TCCGACCTAATTTTTAATGCTTGCCT	56	975
VIC3AVCaV_4R	GACCCTTTCCATTCCCTTTGCC		
VIC3AVCaV_5F	GGAAGAGTTTGGGGCTGTTGAA	62	990
VIC3AVCaV_5R	TCCCTGTCAACCTCGTCCTTAC		
VIC3AVCaV_6F	GCTGTGATAAACAAGGGCCTTG	59	900
VIC3AVCaV_6R	CACCAGCGTACTTTTTGACCCA		
VIC3AVCaV_7F	ACTGCAGGTACATGGAGAAGCA	60	1000
VIC3AVCaV_7R	ATGCAGACTTTGGCTTTGCAGT		
VIC3AVCaV_8F	TGGTGGGAATTTGGATGGTTCAC	59	958
VIC3AVCaV_8R	CTCTGCGACATTCCCAGCAATT		
VIC3AVCaV_9F	GAAGGAGCGGCAAGGAATTTGA	60	972
VIC3AVCaV_9R	GACCTTGCCAGTTTTGGTCAGT		
VIC3AVCaV_10F	GAGGGATTGGGTGAATGCACTG	60	900
VIC3AVCaV_10R	ACCCTCTACAATACTGTGGCCA		
Cherry green ring mottle virus (CGRMV)			
**Primer Name**	**Sequence**	**Tm °C**	**Product size base pair (bp)**
CGRMV_1F	ACCAGAACTAGCACAGATCACCA	60	1020
CGRMV_1R	CATCAGCATTGCCACCCATGAA		
CGRMV_2F	AGGGATCAAATGTTTAGCCATTGTCT	61	879
CGRMV_2R	CTCTGACTGCGTTCCTCCTTCT		
CGRMV_3F	TGCATGACGAGAGGTTCATGAA	62	987
CGRMV_3R	TGCAGCCGAAATGTGAGTGAAA		
CGRMV_4F	CTTGGCTGATGGCGAATCTCTT	61	890
CGRMV_4R	GCTCCCAAATTAGTTGCTGCCT		
CGRMV_5F	AGGCTGAAGTTTGTGATGGCTC	60	974
CGRMV_5R	CATTCCGGCCCATGTTAGCTTT		
CGRMV_6F	AGGACTTCAAAGCTGACTACCTCA	59	920
CGRMV_6R	TCTCCAGTGTCGCCATGGATTT		
CGRMV_7F	CAAGAAGGTAAAGGTGCAGCCA	57	901
CGRMV_7R	TGGAGATCAGCCTCTCACCTAA		
CGRMV_8F	AGGAACGAATACTCATAGCCTTGGA	60	918
CGRMV_8R	GTCTGTTGGTGGTTTGAGGCTC		
CGRMV_9F	GAAATTGTTGGGCCGGATCTGT	59	981
CGRMV_9R	ACCATCTTTGCCTTGTCGCTAG		
CGRMV_10F	GTTTAAATGGCTGATGAGGAGTTTGA	57	934
CGRMV_10R	AGCCAGTCTTCATATTATGCTGGAAG		
LV27S1CGRMV_1F*	ACCAGAACTAGCACAGATCACCA	58	992
LV27S1CGRMV_1R*	TGGTGAAATTGAAACGGCGTCA		
LV27S1CGRMV_2F*	GGTCAGGGATCAAATGTTTAGCCA	61	927
LV27S1CGRMV_2R*	TTTGTCCTCAGGATGCTCGGAT		

LV27S1CGRMV_3F*	GGCTAACTTGGTTAACTCTCTTGCA	61	943
LV27S1CGRMV_3R*	AAGTGAGTGAAACCCGACCAGA		
LV27S1CGRMV_4F*	TGTTGAGGTTAACTTGGCTGATAGTG	62	902
LV27S1CGRMV_4R*	GCTCCCAAATTAGTTGCTGCCT		
LV27S1CGRMV_5F*	GGCTGAAGTTTGTGATGGCTCA	62	1012
LV27S1CGRMV_5R*	AATTCATGCCTGTGCTCTCACC		
LV27S1CGRMV_6F*	AACATTTCTTGTTAGCTCATTCACAGAA	62	898
LV27S1CGRMV_6R*	ACCGCCCACTATGATTCTCCAA		
LV27S1CGRMV_7F*	GGTGAAGGTGCAGCCAAATTTT	59	1009
LV27S1CGRMV_7R*	TGCAACCCTCCCCTTCCTTAAA		
LV27S1CGRMV_8F*	TGTCTGAAAAAGAATTGGATGCTCAC	56	941
LV27S1CGRMV_8R*	TCCCATCTGAGTAATAACCACCGT		
LV27S1CGRMV_9F*	TTGCTGGTGTTTCCTTGGCTTT	59	891
LV27S1CGRMV_9R*	TCAAGGCCGATCCCATTCCTAG		
LV27S1CGRMV_10F*	AGTGTGTTTCAAATGGCTGATGAAG	58	942
LV27S1CGRMV_10R*	AGCCAGTCTTCATATTATGCTGGAA		
LV27S2CGRMV_1F*	ACAAACAGACCAGAACTAGCACA	58	1064
LV27S2CGRMV_1R*	CAGACAGGATGACCTCATGCCT		
LV27S2CGRMV_2F*	TCGTGTCTTCAGTGATTTTGATGCT	61	957
LV27S2CGRMV_2R*	GCATGTGTTGGCGAGTATGGAA		
LV27S2CGRMV_3F*	AGAACTAGGGCAGTGATACGTAACT	59	996
LV27S2CGRMV_3R*	AGAAGCAGTCTCCATCAGCCAT		
LV27S2CGRMV_4F*	AGTATTATGAAGTTATGAATGGGTGCTCC	56	885
LV27S2CGRMV_4R*	AACCAATCAAACTGCCCTGCAA		
LV27S2CGRMV_5F*	CATTCACTCCATCCCTCCCCAT	61	944
LV27S2CGRMV_5R*	TCTGGGCGCAAAACTCTTGAAG		
LV27S2CGRMV_6F*	CTTGTTGACGAAAGGAGGTGGG	60	997
LV27S2CGRMV_6R*	ACCCAGCCCCTTAACTCATCAA		
LV27S2CGRMV_7F*	TGCCGTTTAGCTCCTTATATTAGATACA	60	1014
LV27S2CGRMV_7R*	GATTGAAGAGGGGAAGGAGCCT		
LV27S2CGRMV_8F*	GATCTTGAGGACTTTCACGCCC	60	939
LV27S2CGRMV_8R*	TATCTCACACCCAGCAACCAGT		
LV27S2CGRMV_9F*	ACTGCATTCATCATCGCGTGTT	57	912
LV27S2CGRMV_9R*	AGTGCCTCTCCGGATTTCATCA		
LV27S2CGRMV_10F*	TTAATGGCTGATGAGGAGTTTGAGA	61	946
LV27S2CGRMV_10R*	AAAACCCTACAGCCAGCCTTCA		
Cherry necrotic rusty mottle virus (CNRMV)			
**Primer Name**	**Sequence**	**Tm °C**	**Product size base pair (bp)**
CNRMV_1F	ACAAACCAGTCTAGCACAGATTAACA	62	900
CNRMV_1R	GGACTGAGTAAGGGCCAAGTCT		
CNRMV_2F	GATGGAGTGTGGGCAGAATCTT	56	960
CNRMV_2R	TGCTCCCTCGCAATCTCAATCT		

CNRMV_3F	TCATCCTTGGTGATTCTTGCAAAC	60	992
CNRMV_3R	TTGAGTGTCCTTAACGCCATGC		
CNRMV_4F	TGATCCAAGGAAATTTATGGAAATCACAC	60	934
CNRMV_4R	CACCGCTTGGAGCTTTGAAAGA		
CNRMV_5F	CCAAAGGGCTCCAGACATGCTA	61	1002
CNRMV_5R	TCTTGAGTGAGGAGGACACATGT		
CNRMV_6F	TGTTGTTAAAGCCAACATGGGAAAA	58	977
CNRMV_6R	GAAACAAGCCAAAGTTTGCCCTG		
CNRMV_7F	TGGGAGGTAGAAAAAGCGCTCA	62	996
CNRMV_7R	ACACCGTGAACAACAACAGGAA		
CNRMV_8F	CCAGCCCAGATAGAAACTTTGGT	59	971
CNRMV_8R	TGAGTAGGTCTACTGCACGCAT		
CNRMV_9F	AATTGTGCCAGTGGAACGAGTG	59	967
CNRMV_9R	TAACAAGCCCAGCAACAGGTTG		
CNRMV_10F	AAGATGGCAGATGAGTACGAGCT	57	935
CNRMV_10R	AGCCAGATTCAGGTTAAGCCATG		
Cherry virus A **(CVA)**			
**Primer Name**	**Sequence**	**Tm °C**	**Product size base pair (bp)**
CVA_1F	TTCCAAACACTTTCAAAACTCCCAAA	61	1052
CVA_1R	AGCTTGGCCAAGGATGATTCAG		
CVA_2F	GTGGAGAATGGAAAAATCAAGGGGT	58	959
CVA_2R	GGCAGGAATCCCAATGGTTTCA		
CVA_3F	CATCATCTCAACAATCTGAAGAAAAACCA	62	890
CVA_3R	CAGGTGGGTTCAAGGAGCATTC		
CVA_4F	TGACAAGCTAAAAATCAGCAGGAAGA	56	1004
CVA_4R	GCCCTCATCTCACTGATCTCCA		
CVA_5F	TGCAACAGATTGAAGAAAACTCAATTGA	60	1010
CVA_5R	TAGCACACATATCGTCACCTGC		
CVA_6F	AGTTTTGCACCTTCCTATTCAACACA	60	942
CVA_6R	TCGTCTCCTGCGACTCTTTGAA		
CVA_7F	GGCTCTACCTTAGGTTTAAGAGTTTTGG	58	959
CVA_7R	TGATCAACTGAAAACCTCTGTTCTGG		
CVA_8F	TCCCAAAAACTGTTGGTAAAGATCCT	57	963
CVA_8R	AGTGACCAAGTACACACCCTTGA		
CVA_9F	AGGGACTCAAATCTCCAGCATG	60	948
CVA_9R	GATAGCACTCGAAAGCGGAAAGG		
CVAgrp1_1F*	TCAATTTCCAAATACTTTCAAAACTCCCA	62	1290
CVAgrp1_1R*	CAGCATTGGCAATCCAGTCTCT		
CVAgrp1_2F*	TGACTCTTGGATCATTCAATCTCCC	62	1140
CVAgrp1_2R*	GTCAACACGAACCCCCAGATTC		
CVAgrp1_3F*	GCTCAGGATCAAGTGCTCCAAT	59	1063
CVAgrp1_3R*	TCTCACCTTGTATAGCAGCAGGA		

CVAgrp1_4F*	AGCACCTTCAATAGCGTCATTTTGA	57	1142
CVAgrp1_4R*	AACGCCAAGGTACCTCAAAAGC		
CVAgrp1_5F*	CAGTGGGGTCTGTACAGAGTCA	60	1134
CVAgrp1_5R*	TTGATTCGTCTCCTGCGACTCT		
CVAgrp1_6F*	AGGTTTAAGAGTTTTGGAGGGCTT	56	1127
CVAgrp1_6R*	GCTGGTTTCGCAGTCAGATGTA		
CVAgrp1_7F*	AGCCAGAAGGTATCATGCCAGA	57	1098
CVAgrp1_7R*	TGGAACAAACGATGCAAATCCCA		
CVAgrp2_1F*	CACTTCCATCAACTTCCAAAAACTTCC	59	1182
CVAgrp2_1R*	CTTAGAAAGCTCCAAGGGCTGC		
CVAgrp2_2F*	GTGACTCTTCACCAAGTTCTGATGA	59	1174
CVAgrp2_2R*	CGCTTGATCCTGAACCGTAAGTC		
CVAgrp2_3F*	AGATCCAAAATACCAAAGGATAGCCA	60	1153
CVAgrp2_3R*	ATTTCACTCTGGATGGCAGCAG		
CVAgrp2_4F*	ACAAGGGATTTCTTCTCCAACCC	62	1200
CVAgrp2_4R*	CTCCAGTGAACCGCATTATGGC		
CVAgrp2_5F*	GCTTTTGAGATACCTCGGTGTGT	59	1190
CVAgrp2_5R*	ATTGCTTTCACCAGTGCAGCTT		
CVAgrp2_6F*	GCGAAGGACATTTACAGTGATGC	57	1136
CVAgrp2_6R*	GTCCCCATCACTGCAAGATTCC		
CVAgrp2_7F*	AGAGCTAGAAGATATCATGCCAGGAG	56	1149
CVAgrp2_7R*	AGCACTCAAAAGCGGAAAGGTT		
CVAgrp3_1F*	CCACCAACTTCCAAACACTTCCA	60	1169
CVAgrp3_1R*	ACTCCAAGGACTGCGTTTGTTG		
CVAgrp3_2F*	AGTGATTCATCTCCAAGTTCTGATGA	61	1138
CVAgrp3_2R*	CCAATGAATGATTGTGGCCCCA		
CVAgrp3_3F*	GGTAAACCTGGGCATTTATTTTGCA	60	1189
CVAgrp3_3R*	ATTTCACCCTGGATTGCAGCAG		
CVAgrp3_4F*	GCACTTTCAACAGTGTGATTTTGATTGT	56	1157
CVAgrp3_4R*	TCAATCATGCTGTTGGAAATGCC		
CVAgrp3_5F*	TCTGTACAGAGTCAGATTATGAAGCCT	60	1130
CVAgrp3_5R*	ATTCTTGACTCGTCTCCTGCGA		
CVAgrp3_6F*	AGGTTTAAGAGTTTTAGGGGGCTTG	61	1065
CVAgrp3_6R*	TCCAAGGCCCCTTCTTATCTCG		
CVAgrp3_7F*	AGCTAGAAGATATCATGCCAGAAGC	62	1145
CVAgrp3_7R*	GCACTGAAAGCGGAAAGGTTCT		
CVAgrp4_1F*	TCAACTTCCAAAAACTTCTAAAACTTCCA	60	1264
CVAgrp4_1R*	AGTTCCGTAATTGCTCTGCTGC		
CVAgrp4_2F*	CCCTGGGACTTTCTAAGCGATG	57	1185
CVAgrp4_2R*	CCTTGCGATCTACACGAACACC		
CVAgrp4_3F*	GCATGGGATCTAGTGCTCCAAT	62	1067
CVAgrp4_3R*	CCATCTCGCTTTGAATTGCAGC		

CVAgrp4_4F*	AGACTTTTTCTCAAATCCAATTGAAGCC	57	1192
CVAgrp4_4R*	CAGTGAATCGCATGATGGCCAA		
CVAgrp4_5F*	CTTTTGAGATACCTTGGTGTGTCCA	61	1192
CVAgrp4_5R*	AACATTACTCTCGCCAGTGCAG		
CVAgrp4_6F*	ACAGTGATGCAAATGCGTTCAAT	58	1147
CVAgrp4_6R*	GGATAATCCGTCTGCTCGCTTG		
CVAgrp4_7F*	AAGAGCTCGGAGGTATCATGCC	61	1070
CVAgrp4_7R*	ACCAGGGAAGAGAAACTAACCTCTC		
Little cherry virus 1 **(LCHV1)**			
**Primer Name**	**Sequence**	**Tm °C**	**Product size base pair (bp)**
LCV1_1F	TCCCAGCTTTGTGCCTAAGAGA	57	936
LCV1_1R	TATCCTCAAAGGGACGGGTGTT		
LCV1_2F	GCTGGTGTCAATTCAAAATTACCTAGG	58	999
LCV1_2R	AATGGTAGAGTGCATCCCCAGT		
LCV1_3F	TTGTCCCAAACTTGAACGTTGGT	59	896
LCV1_3R	TCAAATCATGGGTGTATGCTTCTGC		
LCV1_4F	TTCAATACAATAACAATATTCGAAGGTGGT	60	889
LCV1_4R	GACAACTCTGTCGCAGTGTCAT		
LCV1_5F	GCTGATGGCGACGTTCATTCAA	59	901
LCV1_5R	ACAAGTCTAGACGCTCCAAAATGT		
LCV1_6F	TGGTCGATGAAGATCTCAATGATGC	58	920
LCV1_6R	AATGTATCTCCAGTGGCAATGGC		
LCV1_7F	TCAATCCATTCGCAGCAATTTTCTT	60	944
LCV1_7R	TTTTGTTTGAAAATCCCGCGGC		
LCV1_8F	TGAGTACTCTGTTACATCCAGTCGT	62	961
LCV1_8R	TCGTTCATAGCATTAGAGGGGCA		
LCV1_9F	CAGGTTGAGATTGCATAATAATACGCT	61	922
LCV1_9R	CCCATCTCATCAAGGTTCATGCC		
LCV1_10F	TGTTTTTCAGCCCAATCTTTCTGGA	58	882
LCV1_10R	GCCGAGAAAACACAAACCCAAAC		
LCV1_11F	GTGGTTACGCTGCTTTTTGCTT	61	958
LCV1_11R	ACTCCAGGTGTCACAGATTCCTT		
LCV1_12F	CGGTGGTAGAGATATTGACAGGC	56	997
LCV1_12R	ACAACAATCGAACTACCAAGCGT		
LCV1_13F	TTCATTGATAATAATGGAGGTCTTGACGA	58	948
LCV1_13R	GCGTATAACTTATCCCTTTCTTCTACTGG		
LCV1_14F	TGATCAACCTGACATATTGACTAAGTGT	61	1033
LCV1_14R	CCATCGTAAAAACTCTGTTCTCAAAACT		
LCV1_15F	TTGATTATTTGTTGAAACCCACATAGAGT	60	963
LCV1_15R	ACTGGTCCTCGTTGCCATTCAT		
LCV1_16F	ACGAGCAATCTACCACGACTAAGT	58	984
LCV1_16R	AGCGATTGATTTATGCGACGGT		

LCV1_17F	AGTCGGCGAACCACACTATCAA	61	972
LCV1_17R	GGCCAATGTCGACTGTTGCTTTA		
LCV1_18F	AGACCGAGAGTAGAGTCAGAGAGT	56	935
LCV1_18R	ACCAGTGATTTTAGTGGAATTCGCA		
LCV1_19F	CGCGGAAAGACGAAACATTAGAGA	59	946
LCV1_19R	TCAGTCGCTATGAGATTTCGGGAT		
LCV1_20F	ACGGCGAATTAGCTTACAATTTGGT	56	945
LCV1_20R	TGCATCTTAGGTGGTTTATCTTAACAGAT		
LV27S1LCV1_1F*	TGCATACCTTAAGCTTTTGTGCCT	58	1036
LV27S1LCV1_1R*	CCCAACATCGATCAGGTGGTTG		
LV27S1LCV1_2F*	TGAGGACATTGTTGATGCTCTGAA	62	966
LV27S1LCV1_2R*	TTCGTTCATTGCAGACCCAACT		
LV27S1LCV1_3F*	GCATAAGACCGGTAATGCATTGTATC	58	884
LV27S1LCV1_3R*	GCTACGCCATCAATGCTAACGA		
LV27S1LCV1_4F*	GGCGACAAGGTCTATTATTATTACGGT	61	881
LV27S1LCV1_4R*	TAAATCAGAGACGCCCATGCCT		
LV27S1LCV1_5F*	AGGTGTGCAGATCTAGAGCAGC	62	930
LV27S1LCV1_5R*	TCCGCACCTTAACTTCCTCGAA		
LV27S1LCV1_6F*	AACTACATCTTAGATCGCTTAAACTTGTTT	60	884
LV27S1LCV1_6R*	TGCCTTCTTCAAAGCTCCAACA		
LV27S1LCV1_7F*	ACAAAACTTTTTATGATGGCATTGTTCG	57	890
LV27S1LCV1_7R*	AAAATCCTGCGGCAAACTCAGT		
LV27S1LCV1_8F*	ACTGTTGAGTTTTCTGTAACCTCTGA	59	969
LV27S1LCV1_8R*	GCTGTCATTCATAGCATTTGACGG		
LV27S1LCV1_9F*	AGCTTCAGGGAGGTACCTTCAA	60	980
LV27S1LCV1_9R*	ACGACTTTCGAGGAGATCTCCC		
LV27S1LCV1_10F*	AGTCCAATTTTTCTTGAAATTTTTGGAAGG	57	985
LV27S1LCV1_10R*	CGTCGAGAACGTAGTCCCAAAA		
LV27S1LCV1_11F*	GGTTGTTTGTTTCTTTATCTCTCCTTCC	62	1012
LV27S1LCV1_11R*	CCAGCATTGATGTTCCACCTACC		
LV27S1LCV1_12F*	ACTCGGTAGCTCAACCTTTCATTG	59	882
LV27S1LCV1_12R*	ACAAGCTTACCTGTGAAACACCT		
LV27S1LCV1_13F*	TTAGATGAACGCTTGGTAGTTCGA	59	945
LV27S1LCV1_13R*	TCAAACTGTCCTTCTCGTTGATGT		
LV27S1LCV1_14F*	TGTAGTCTGATATATTTGCTACTACCGGT	62	949
LV27S1LCV1_14R*	TGAATGCTGTCTCTGCCATCAA		
LV27S1LCV1_15F*	TTTGATTATTTGATGAAACCCACATAGAGT	56	960
LV27S1LCV1_15R*	GCCACGTTGCCATTCGTACTTA		
LV27S1LCV1_16F*	ACTTCTTTGTATGAGCAATCTACTACCA	57	978
LV27S1LCV1_16R*	GCAGGTCCGTCACTCAAATACC		
LV27S1LCV1_17F*	GGGGTTAAACTGTTGAATATCAAGCC	56	954
LV27S1LCV1_17R*	TTGGTCTAGACTGAGGCGTGTT		

LV27S1LCV1_18F*	TGACTAATCACCATATCAACAATAACGTTT	58	902
LV27S1LCV1_18R*	ACTGCGCAATTCGTTCATGGAA		
LV27S1LCV1_19F*	AGAGAGTTGAAACTTTCGCTACTCA	56	958
LV27S1LCV1_19R*	CATGTTTCCATCGTCCCAGCTC		
LV27S1LCV1_20F*	TGTGGTATATTGAACGGTGAATTAGCT	57	875
LV27S1LCV1_20R*	AGTTTATGACTAGAGAAGGTAAGCGGT		
LV27S2LCV1_1F*	CTGCATACCTCCCAGCTTTGTG	62	1037
LV27S2LCV1_1R*	CCCAACATCGATCAGGTGGTTG		
LV27S2LCV1_2F*	TGAGGACATTGTTGATGCTCTGAA	61	884
LV27S2LCV1_2R*	GCGTCGCCAGTCTTGTGTAAAA		
LV27S2LCV1_3F*	CCTACATTTGTTCCGTTTATCCCCA	61	899
LV27S2LCV1_3R*	AGCCTCAGCAGCATCTCCATAA		
LV27S2LCV1_4F*	ACCAGGTGATCTTCTTGAAGATTTTAATAC	61	933
LV27S2LCV1_4R*	ACCTCCTGAATCGACAGCATCA		
LV27S2LCV1_5F*	ACAGAAAATTTTGAGAAGTACCAACAAAGA	58	881
LV27S2LCV1_5R*	ACACTTGAATAAGTCCAAACGACCT		
LV27S2LCV1_6F*	TGGTTGATGAAGATCTTAATGAAGCTCT	62	924
LV27S2LCV1_6R*	AGCAAAGAATCCCCAGTTGCAA		
LV27S2LCV1_7F*	GCAGCTATTTTCTTCTTTGTAGCAGC	61	926
LV27S2LCV1_7R*	TGAGAAACCCGCTGCAAATTCA		
LV27S2LCV1_8F*	ACGTGCCTCAAACGATAGAGTAC	60	938
LV27S2LCV1_8R*	CAGTATGCCTCGAAATAGCCGT		
LV27S2LCV1_9F*	GGACTGTTAATGAACTCCAAGGTGG	58	991
LV27S2LCV1_9R*	ACGACTAGTTAGAAGATCGCCCA		
LV27S2LCV1_10F*	TCCAATTTTTCTTGAAATTTTTGGTCGGA	60	905
LV27S2LCV1_10R*	CAACGTTGGAAGGCGAAACAAA		
LV27S2LCV1_11F*	TGGTAGTGGTTACGCTGCTTTT	58	917
LV27S2LCV1_11R*	GCAAGAGTGGCCAGTCTTTTCA		
LV27S2LCV1_12F*	TGACGTATCTTACATTAAGAAACATGGGA	62	903
LV27S2LCV1_12R*	TGCCCCAAAACTTTGACAATGATAGT		
LV27S2LCV1_13F*	TGACGGTGGGAAAGTGAAGGAA	61	986
LV27S2LCV1_13R*	TCAGTGTATCTCTCGTCTAACACCA		
LV27S2LCV1_14F*	TCGGTAACTTAGACAATTCAGATCTGT	60	895
LV27S2LCV1_14R*	CCTCTCATTCACACGTATGGCC		
LV27S2LCV1_15F*	AAGTCTAAGCACTTTGTTGGATACATCG	60	980
LV27S2LCV1_15R*	TTCAAACGGCTAGTGGGTTCCT		
LV27S2LCV1_16F*	GAACTTCAACGAAACCAGTTTAAAAACA	56	958
LV27S2LCV1_16R*	CGATTGCTCGTTATTCAACAAGCTG		
LV27S2LCV1_17F*	ACGTATTATGTAATGGTGGGTAACGA	58	933
LV27S2LCV1_17R*	AGGTCGCTCTTTACCATACAAGC		
LV27S2LCV1_18F*	CACAGTTTTACAAAGGATTATAGTTCAGGA	57	971
LV27S2LCV1_18R*	CACTTCGTGCTCTTGGGTTGTT		

LV27S2LCV1_19F*	AGGTAGGGCAATTGTTTTGGGA	57	968
LV27S2LCV1_19R*	ACAGTGTCCATGCTGTTTGAGA		
LV27S2LCV1_20F*	GGTGAATTAGCTTATAGTTTAGTTGTTGGT	59	876
LV27S2LCV1_20R*	AAACACTCAGTTAAAACCAGTTTATGACT		
Little cherry virus 2 (LCHV2)			
**Primer Name**	**Sequence**	**Tm °C**	**Product size base pair (bp)**
LCV2_1F	TCCCTTTGTTGTTTGTCTCAAGTCT	56	889
LCV2_1R	CCTGCGGACAACCACCAATATC		
LCV2_2F	TCCAGATCCGAACACCCAATGT	57	883
LCV2_2R	CGAGAGCCAACGATCAGATGTG		
LCV2_3F	ATCATACCTGTTGAACGTGGCG	56	958
LCV2_3R	TGTCCCAATTTGAAACCCTGCG		
LCV2_4F	AAAGGGGATTTTGGGATGCGAC	57	951
LCV2_4R	ATGACACGTAACCCCCAAGAGT		
LCV2_5F	CGTCGTTTTACCTTGGGTGGAA	57	1007
LCV2_5R	GCGTGTGTGATTAGCTCCGATT		
LCV2_6F	CCTGCTGGGATAAAAACACTTTTCA	57	953
LCV2_6R	TGTCTTCCCCTGGATCTCATGG		
LCV2_7F	ACTTTCACACAAGATGAGAAACATGC	62	1001
LCV2_7R	CGGTGAAGCCTCTCAACTGTTT		
LCV2_8F	CTGTGTTGTCTAGCATACCTAGTGG	57	899
LCV2_8R	ACTCGAAACCAAATGCCACGTT		
LCV2_9F	AACATGTCTACGACGCAGCTTG	58	997
LCV2_9R	GCGAACCAAGAACCGACCATTA		
LCV2_10F	GGGCACATCTTATTACAAGTTTGGTATG	58	897
LCV2_10R	GCTGCCTCTCCTGTTGTGTGTA		
LCV2_11F	CAGAACAAGGCACGGTATTGGT	58	985
LCV2_11R	ACCAGATAACGCAACGGTGAAA		
LCV2_12F	ACGTTGGGTAGGTGTGAACAAA	61	1013
LCV2_12R	CCGTAGTTCACCTCAGCAGTTG		
LCV2_13F	TCACACGCAATTTTGGACGAGG	57	969
LCV2_13R	AGTCTACCGCCTTTCTACACCT		
LCV2_14F	TCTTTGGTGGAAATAAGTGAGGCA	57	903
LCV2_14R	CACTTAAACGATCGGCCGGTAA		
LCV2_15F	GCAAAAACAGGGTTCTTACTTCTGG	61	970
LCV2_15R	AGAATTCCACTGGTCTACGGGT		
LCV2_16F	TGAAACATGACTACATCATCCAAGGA	58	933
LCV2_16R	AACCGTCTCTACCTGCATCTGG		
LCV2_17F	TTGACCAGTGGGATGATAAAACCC	57	1001
LCV2_17R	ATAGAACTAACGATGGCGCTGC		
LCV2_18F	TGTCGTCGAAAACAATACACAATGC	58	936
LCV2_18R	TGTCGTAGGGTACTTACATACTATTCTAGA		

Nectarine stem pitting-associated virus (NSPaV)			
**Primer Name**	**Sequence**	**Tm °C**	**Product size base pair (bp)**
NSPaV_1F	ACTGACTTTACAAGACTGGGGCT	59	1023
NSPaV_1R	GGACAACCATCGCCATGTCAAT		
NSPaV_2F	ACTTCTCTCTGGAGGCCAAGAC	59	895
NSPaV_2R	CATCACCGAACACCGCATTGTA		
NSPaV_3F	TCCTGTGGCAATTGGAGTTGATG	62	1003
NSPaV_3R	GGTTGTTGTCGCATTGGTGGTA		
NSPaV_4F	GCTTAAGCACCTGACTAACTGCT	60	1024
NSPaV_4R	GTACGCTACACAACCCAACCAA		
NSPaV_5F	CCAGGTTCTTTTTCAGTATACGTGGA	56	883
NSPaV_5R	ATTTTCCATCGGCCACACACAA		
NSPaV_6F	GGCTCCGTTTCCATATCTGTGG	62	893
NSPaV_6R	GAGCCCATGGGATTTTAAGCCT		
Plum bark necrosis stem pitting associated virus (PBNSPaV)			
**Primer Name**	**Sequence**	**Tm °C**	**Product size base pair (bp)**
PBNSPaV_1F	CTAGCGCCTGCGAGATAAATCC	60	893
PBNSPaV_1R	TCAGAGCCCAAATCAGACCACT		
PBNSPaV_2F	GCAGGGAGGTTTTGGATCTCAA	57	917
PBNSPaV_2R	AAGCAACATCCCTCCCATCCTT		
PBNSPaV_3F	GGCTTCACACTCTTCTGTATCACC	59	900
PBNSPaV_3R	GTGAGTGCTCTGCTTCAGCTTC		
PBNSPaV_4F	TCAAGACCTGTTTTTAAGGAAGGCTC	59	880
PBNSPaV_4R	CGCCAAGCAGGAAAAACCCTAG		
PBNSPaV_5F	TGGAATTAAGTGGTTGTTATGGACCA	62	955
PBNSPaV_5R	CCAAGTCGAAGTAAGCAGGCTG		
PBNSPaV_6F	TCTTCTAAAGAAAGTCTTTTCCTCGGC	57	953
PBNSPaV_6R	GTTGCCAAATTCTCCACAGGCT		
PBNSPaV_7F	GGCTTCTCTTGATGGTGATAAGGC	62	897
PBNSPaV_7R	ACCAGAACATCGCAACAGTTCC		
PBNSPaV_8F	TGTTTCTGAAGCTAGCTCTTTCTCA	60	1004
PBNSPaV_8R	ATATCAGCCGGACACCTGTAGG		
PBNSPaV_9F	ACCCTTCATTCCTCGAACTGAAG	57	995
PBNSPaV_9R	CACCCACAACCTCCGAAAACTT		
PBNSPaV_10F	GAGACCAATCGCTCATTACGACC	60	907
PBNSPaV_10R	ACAGGGTCAGGGACAAACTGAA		
PBNSPaV_11F	TTGCTTTCGATGTTAAGCTCTTCAG	57	975
PBNSPaV_11R	AATCTGCGGGGACACTACAAGA		
PBNSPaV_12F	CGTCATGCCTGTTGTGGATTTGA	61	916
PBNSPaV_12R	CCTTCATGAAGAACAAAGGCCGT		
PBNSPaV_13F	AGCCCTTTTATGCAAAGTTGGTGT	60	930
PBNSPaV_13R	CCTCAGTACAAAGCCTTCCGAG		

PBNSPaV_14F	TCCAAGATTTTTCTTACACAGTCGACA	62	916
PBNSPaV_14R	CTCTGTTGCATAACCTCCAGCC		
PBNSPaV_15F	ACTATACCTCGTAGATTTCAGGGGG	56	1007
PBNSPaV_15R	CAGGTGCAGCTGTTTTCACTGT		
PBNSPaV_16F	GTGGGCGTTGAGTAAAGGACTG	60	955
PBNSPaV_16R	ATGCCATCCAAAACATACCCCC		
PBNSPaV_17F	GGGTCGTATGGTGGCATTGAAT	59	962
PBNSPaV_17R	CCCGGGCAAGAAATGTGTGAAT		

*Primers pairs used to amplify and confirm full genome of multiple viruses assembled within a *Prunus* tree sample.

Table S3. The GenBank accession numbers, country of origin and host species of full genome virus isolates of *Apple chlorotic leafspot virus* (ACLSV), *Apricot latent virus* (ApLV), *Apricot pseudo-chlorotic leaf spot virus* (APCLSV), *Apricot vein clearing-associated virus* (AVCaV), *Asian prunus virus* (APV2), *Cherry virus A* (CVA), *Cherry green ring mottle virus* (CGRMV), *Cherry necrotic rusty mottle virus* (CNRMV), *Little cherry virus 1* (LChV1), *Little cherry virus 2* (LChV2), *Nectarine stem pitting-associated virus* (NSPaV) and *Plum bark necrosis stem pitting-associated virus* (PBNSPaV) that were used in the phylogenetic analysis

**ACLSV accession**	**Country**	**Host**	**CVA accession**	**Country**	**Host**
M58152	France	Plum	FN691959	India	Cherry
KX579123	Germany	Apple	KU131205	China	Cherry
KX579122	Germany	Apple	KU215410	Czech	Cherry
KU960942	China	Apple	KU215411	Czech	Cherry
KU870525	China	Hawthorn	KX370827	China	Cherry
KU870524	China	Hawthorn	KY286055	South	Apricot
KC935956	China	Pear	KY445749	South	Apricot
JN634761	China	Pear	KY510845	Canada	Cherry
JN634760	China	Pear	KY510847	Canada	Cherry
AB326225	Japan	Apple	KY510858	Canada	Cherry
AB326224	Japan	Apple	KY510859	Canada	Cherry
AB326223	Japan	Apple	KY510863	Canada	Cherry
D14996	Japan	Apple	KY510864	Canada	Cherry
MF069041	Czech	Cherry	KY510865	Canada	Cherry
KY310578	South Korea	Peach	KY510866	Canada	Cherry
KY310577	South Korea	Peach	KY510873	Canada	Apricot
KY310576	South Korea	Peach	KY510875	Canada	Apricot
KY310575	South Korea	Peach	KY510876	Canada	Apricot
KX506849	South Korea	Apple	KY510880	Canada	Apricot
KJ522693	China	Apple	KY510881	Canada	Cherry
AJ243438	Germany	Plum	KY510885	Canada	Cherry
X99752	France	Plum	KY510886	Canada	Cherry
EU223295	USA	Peach	KY510891	Canada	Cherry
**APCLSV accession**	**Country**	**Host**	KY510892	Canada	Cherry
AY713379	Italy	Peach	KY510906	Canada	Cherry
KY310579	South Korea	Peach	KY510909	Canada	Cherry

**ApLV accession**	**Country**	**Host**	KY510911	Canada	Cherry
HQ339959	Italy	Apricot	KY510912	Canada	Cherry
HQ339958	France	Apricot	KY510916	Canada	Cherry
HQ339957	France	Peach	KY510918	Canada	Cherry
HQ339956	Italy	Apricot	KY510919	Canada	Plum
**APV2 accession**	**Country**	**Host**	LC125634	Japan	Apricot
KY445748	South	Korea	**LChV1 accession**	**Country**	**Host**
KR998049	USA	Peach	EU715989	Italy	Cherry
KR998048	USA	Peach	MG934545	Japan	Cherry
KT893295	Japan	Apricot	KX192367	Spain	Cherry
KT893294	Japan	Apricot	KX192366	Spain	Cherry
KY310582	South Korea	Peach	KR736335	China	Cherry
KY310581	South Korea	Peach	LN794218	Greece	Cherry
**AVCaV accession**	**Country**	**Host**	KR080325	South Korea	Peach
KY132099	Australia	Plum	Y10237	Germany	Cherry
KM507062	France	Plum	MK895512	Belgium	Cherry
HG008921	Italy	Apricot	MK895511	Belgium	Cherry
KM507063	France	Plum	MH300061	Spain	Cherry
**CGRMV accession**	**Country**	**Host**	MH300060	Spain	Cherry
AF017780	USA	Cherry	**LChV2 accession**	**Country**	**Host**
JX501671	China	Peach	AF531505	USA	Cherry
JX501670	China	Peach	MG881767	China	Cherry
KY178277	Germany	Cherry	MK895513	Belgium	Cherry
KY178276	Moldova	Cherry	MK803502	Belgium	Cherry
KY178275	USA	Cherry	MF069043	Germany	Cherry
KR820548	China	Peach	MF326520	South Korea	Peach
KC218931	USA	Cherry	KT273410	USA	Nectarine
AJ291761	France	Cherry	KT273409	USA	Nectarine
**CNRMV accession**	**Country**	**Host**	KP638562	USA	Nectarine
EU188439	Japan	Cherry	**PBNSPaV accession**	**Country**	**Host**
EU188438	Japan	Cherry	EF546442	USA	Plum
KY178274	Moldova	Cherry	KU240013	China	Cherry
KY310583	South Korea	Peach	KC590347	China	Plum
AF237816	Germany	Unknown	KC590346	France	Plum
KR820549	China	Cherry	KC590345	France	Plum
KF030832	USA	Cherry	KC590344	China	Peach
			KJ792854	China	Peach
			KJ792853	China	Plum
			KJ792852	China	Peach
			HG917400	Italy	Apricot

Table S4. The viruses and viroids that were detected by RT-PCR and High throughput sequencing (HTS) testing during the survey of 100 and 24 Australian *Prunus* trees respectively, known to infect *Prunus* species. The table also lists viruses that were not been previously reported in Australia and were not detected during the survey and currently considered exotic to Australia.

**Viruses and viroids known to occur in Australia**	**Genus**	**Tested by RT-PCR in this survey**	**Detected by RT-PCR**	**Detected by HTS**	***Prunus* host in Australia**
American plum line pattern virus (APLPV)	*Ilarvirus*	Yes	No	NT	N/A
Apple chlorotic leaf spot virus (ACLSV)	*Trichovirus*	Yes	13	6	Apricot, Cherry Nectarine, Peach, Plum
Apple mosaic virus (ApMV)	*Ilarvirus*	Yes	1	NT	Almond
Apple stem grooving virus (ASGV)	*Capillovirus*	Yes	1	NT	Plum
Apple stem pitting associated virus (ASPV)	*Foveavirus*	Yes	No	NT	N/A
Apricot latent virus (ApLV)	*Foveavirus*	Yes	No	1	Apricot
Apricot pseudo-chlorotic leaf spot virus (APCLSV)	*Trichovirus*	Yes	2	2	Nectarine, Plum
Apricot vein clearing associated virus (ACVaV)	*Prunevirus*	No	N/A	2	Plum
Asian Prunus virus 2 (APV2)	*Foveavirus*	Yes	No	1	Apricot
Carnation ringspot virus (CRSV)	*Dianthovirus*	NT	NT	NT	N/A
Cherry green ring mottle virus (CGRMV)	*Robigovirus*	Yes	4	7	Cherry, Peach
Cherry necrotic rusty mottle virus (CNRMV)	*Robigovirus*	Yes	2	4	Cherry
Cherry virus A (CVA)	*Capillovirus*	Yes	14	14	Apricot, Cherry, Plum
Citrus enation virus (CVEV)	*Enamovirus*	NT	NT	NT	N/A
Cucumber mosaic virus (CMV)	*Cucumovirus*	Yes	No	NT	N/A
Hop stunt viroid (HSVd)	*Hostuviroid*	Yes	9	NT	Almond, Apricot, Nectarine, Plum
Little cherry virus 1 (LChV1)	*Velarivirus*	Yes	No	5	Cherry, Plum
Little cherry virus 2 (LChV2)	*Ampelovirus*	Yes	5	6	Cherry
Nectarine stem pitting-associated virus (NSPaV)	*Luteovirus*	NT	NT	2	Peach
Peach latent mosaic viroid (PLMVd)	*Pelamoviroid*	Yes	6	NT	Nectarine, Peach
Plum bark necrosis stem pitting associated virus (PBNSPaV)	*Ampelovirus*	Yes	4	7	Cherry, Plum
Prune dwarf virus (PDV)	*Ilarvirus*	Yes	3	NT	Cherry, Cherry, Plum, Peach

Prunus necrotic ringspot virus (PNRSV)	*Ilarvirus*	Yes	52	NT	Almond, Apricot, Cherry, Nectarine, Peach, Plum
Sowbane mosaic virus (SoMV)	*Sobemovirus*	NT	NT	NT	N/A
Tobacco mosaic virus (TMV)	*Tobamovirus*	NT	NT	NT	N/A
Tobacco necrosis virus (TNV)	*Necrovirus*	NT	NT	NT	N/A
**Viruses and viroids not known to occur in Australia**	**Genus**	**Tested in this survey**	**Detected by RT-PCR**	**Detected by HTS**	***Prunus* host in Australia**
Apple scar skin viroid (ASSV)	*Apscaviroid*	Yes	No	NT	N/A
Apricot latent ringspot virus (ALRSV)	*Nepovirus*	No	N/A	N/A	N/A
Asian Prunus virus 1 (APV1)	*Foveavirus*	Yes	No	NT	N/A
Asian Prunus virus 3 (APV3)	*Foveavirus*	Yes	No	NT	N/A
Arabis mosaic virus (ArMV)	*Nepovirus*	Yes	No	NT	N/A
Carnation Italian ringspot virus (CIRV)	*Tombusvirus*	No	N/A	N/A	N/A
Caucasus prunus virus (CPrV)	*Prunevirus*	No	N/A	N/A	N/A
Cherry associated ovirus (ChALV)	*Luteovirus*	No	N/A	N/A	N/A
Cherry leaf roll virus (CLRV)	*Nepovirus*	Yes	No	NT	N/A
Cherry mottle leaf virus (CMLV)	*Trichovirus*	Yes	No	NT	N/A
Cherry rasp leaf virus (CRLV)	*Cheravirus*	Yes	No	NT	N/A
Cherry rosette virus (ChRV)	*Nepovirus*	No	N/A	N/A	N/A
Cherry rusty mottle virus (CRMV)	*Robigovirus*	Yes	No	NT	N/A
Cherry twisted leaf associated virus (CTLaV)	*Robigovirus*	Yes	No	NT	N/A
Epirus cherry virus (EpCV)	*Ourmiavirus*	No	N/A	N/A	N/A
Myrobalan latent ringspot virus (MLRSV)	*Nepovirus*	No	N/A	N/A	N/A
Nectarine virus M (NeVM)	*Marafivirus*	No	N/A	N/A	N/A
Peach chlorotic mottle virus (PCMV)	*Foveavirus*	No	N/A	N/A	N/A
Peach enation virus (PEV)	*Nepovirus*	No	N/A	N/A	N/A
Peach mosaic virus (PcMV)	*Trichovirus*	Yes	No	NT	N/A
Peach rosette mosaic virus (PRMV)	*Nepovirus*	Yes	No	NT	N/A
Petunia asteroid mosaic virus (PeAMV)	*Tombusvirus*	No	N/A	N/A	N/A

Plum pox virus (PPV)	*Potyvirus*	Yes	No	NT	N/A
Prunus virus F (PrVF)	*Fabavirus*	No	N/A	N/A	N/A
Prunus virus T (PrVT)	*Tepovirus*	No	N/A	N/A	N/A
Raspberry ringspot virus (RpRSV)	*Nepovirus*	Yes	No	NT	N/A
Stocky prune virus (StPV)	*Cheravirus*	No	N/A	N/A	N/A
Strawberry latent ringspot virus (SLRSV)	*Unassigned*	Yes	No	NT	N/A
Tobacco ringspot virus (TRSV)	*Nepovirus*	Yes	No	NT	N/A
Tomato black ring virus (TBRV)	*Nepovirus*	Yes	No	NT	N/A
Tomato ringspot virus (ToRSV)	*Nepovirus*	Yes	No	NT	N/A
Tomato bushy stunt virus (TBSV)	*Tombusvirus*	Yes	No	NT	N/A

Table S5. The isolate ID, *Prunus* host, origin and the GenBank accession number of full genome sequences of viruses isolates of *Apple chlorotic leafspot virus* (ACLSV), *Apricot latent virus* (ApLV), *Apricot pseudo-chlorotic leaf spot virus* (APCLSV), *Apricot vein clearing-associated virus* (AVCaV), *Asian prunus virus 2* (APV2), *Cherry virus A* (CVA), *Cherry green ring mottle virus* (CGRMV), *Cherry necrotic rusty mottle virus* (CNRMV), *Little cherry virus 1* (LChV1), *Little cherry virus 2* (LChV2), *Nectarine stem pitting-associated virus* (NSPaV) and *Plum bark necrosis stem pitting-associated virus* (PBNSPaV) detected by metagenomic high throughput sequencing in this study.

**Isolate**	***Prunus* sp.**	**Origin**	**Virus species**	**GenBank accession number**
TAS1	Cherry (*P. avium*)	Tasmania	ACLSV	LC522979
LV27	Cherry (*P. avium*)	Tasmania	ACLSV	LC522976
LV35	Cherry (*P. avium*)	Tasmania	ACLSV	LC522977
LVV	Cherry (*P. avium*)	Tasmania	ACLSV	LC522978
VIC10	Cherry (*P. avium*)	Victoria	ACLSV	LC522980
WA1	Peach (*P. persica*)	Western Australia	ACLSV	LC522981
VIC18	Apricot (*P. armeniaca*)	Victoria	ApLV	LC522982
VIC3	Plum (*P. domestica*)	Victoria	APCLSV	LC522983
VIC11	Nectarine (*P. persica var. nucipersica*)	Victoria	APCLSV	LC522984
TAS10	Apricot (*P. armeniaca*)	Tasmania	APV2	LC522985
QLD2	Plum (*P. domestica*)	Queensland	AVCaV	LC522987
VIC3	Plum (*P. domestica*)	Victoria	AVCaV	LC522986
TAS12	Cherry (*P. avium*)	Tasmania	CGRMV	LC522993
TAS16	Cherry (*P. avium*)	Tasmania	CGRMV	LC522994
LV16	Cherry (*P. avium*)	Tasmania	CGRMV	LC522988
LV27S1	Cherry (*P. avium*)	Tasmania	CGRMV	LC522989
LV27S2	Cherry (*P. avium*)	Tasmania	CGRMV	LC522990
LV35	Cherry (*P. avium*)	Tasmania	CGRMV	LC522991
LVV	Cherry (*P. avium*)	Tasmania	CGRMV	LC522992
VIC5	Peach (*P. persica*)	Victoria	CGRMV	LC522995
LV27	Cherry (*P. avium*)	Tasmania	CNRMV	LC522996
LV35	Cherry (*P. avium*)	Tasmania	CNRMV	LC522997
LVV	Cherry (*P. avium*)	Tasmania	CNRMV	LC522998
QLD11	Cherry (*P. avium*)	Queensland	CVA	LC522999
TAS1	Cherry (*P. avium*)	Tasmania	CVA	LC523000
TAS2S1	Cherry (*P. avium*)	Tasmania	CVA	LC523001
TAS2S2	Cherry (*P. avium*)	Tasmania	CVA	LC523002
TAS4S1	Cherry (*P. avium*)	Tasmania	CVA	LC523003
TAS4S2	Cherry (*P. avium*)	Tasmania	CVA	LC523004
TAS5S1	Cherry (*P. avium*)	Tasmania	CVA	LC523005
TAS5S2	Cherry (*P. avium*)	Tasmania	CVA	LC523006
TAS12S1	Cherry (*P. avium*)	Tasmania	CVA	LC523007

TAS12S2	Cherry (*P. avium*)	Tasmania	CVA	LC523008
TAS12S3	Cherry (*P. avium*)	Tasmania	CVA	LC523009
LV16	Cherry (*P. avium*)	Tasmania	CVA	LC523010
LV27	Cherry (*P. avium*)	Tasmania	CVA	LC523011
LV35S1	Cherry (*P. avium*)	Tasmania	CVA	LC523012
LV35S2	Cherry (*P. avium*)	Tasmania	CVA	LC523013
LVV	Cherry (*P. avium*)	Tasmania	CVA	LC523014
VIC10	Cherry (*P. avium*)	Victoria	CVA	LC523015
VIC12	Plum (*P. domestica*)	Victoria	CVA	LC523016
VIC18	Apricot (*P. armeniaca*)	Victoria	CVA	LC523017
WA2	Apricot (*P. armeniaca*)	Western Australia	CVA	LC523018
QLD13	Plum (*P. domestica*)	Queensland	LChV1	LC523019
TAS16	Cherry (*P. avium*)	Tasmania	LChV1	LC523020
LV16	Cherry (*P. avium*)	Tasmania	LChV1	LC523021
LV27S1	Cherry (*P. avium*)	Tasmania	LChV1	LC523022
LV27S2	Cherry (*P. avium*)	Tasmania	LChV1	LC523023
LVV	Cherry (*P. avium*)	Tasmania	LChV1	LC523024
TAS12	Cherry (*P. avium*)	Tasmania	LChV2	LC523025
TAS16	Cherry (*P. avium*)	Tasmania	LChV2	LC523026
LV16	Cherry (*P. avium*)	Tasmania	LChV2	LC523027
LV27	Cherry (*P. avium*)	Tasmania	LChV2	LC523028
LV35	Cherry (*P. avium*)	Tasmania	LChV2	LC523029
LVV	Cherry (*P. avium*)	Tasmania	LChV2	LC523030
TAS17	Peach (*P. persica*)	Tasmania	NSPaV	LC523031
VIC5	Peach (*P. persica*)	Victoria	NSPaV	LC523032
QLD2	Plum (*P. domestica*)	Queensland	PBNSPaV	LC523033
QLD13	Plum (*P. domestica*)	Queensland	PBNSPaV	LC523034
TAS12	Cherry (*P. avium*)	Tasmania	PBNSPaV	LC523035
LV27	Cherry (*P. avium*)	Tasmania	PBNSPaV	LC523036
LVV	Cherry (*P. avium*)	Tasmania	PBNSPaV	LC523037
VIC2	Plum (*P. domestica*)	Victoria	PBNSPaV	LC523038
VIC3	Plum (*P. domestica*)	Victoria	PBNSPaV	LC523039

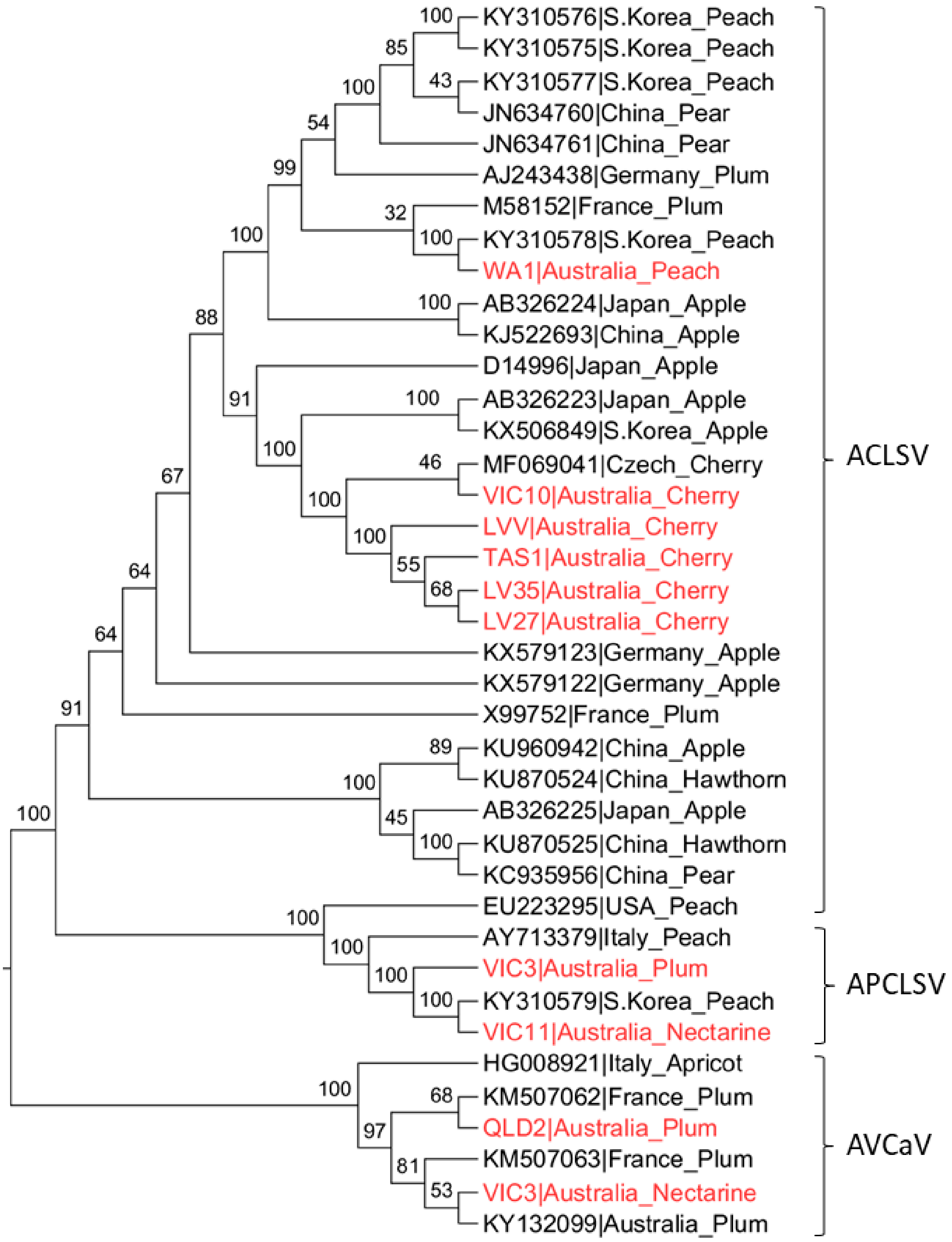

Figure S1. Maximum likelihood phylogenetic tree of full-length genome sequences of Australian *Apple chlorotic leafspot virus* (ACLSV), *Apricot pseudo-chlorotic leaf spot virus* (APCLSV), *Apricot vein clearing associated virus* (AVCaV) isolates and the corresponding GenBank isolates of each virus (Table S3). The phylogenetic tree was constructed using MEGA (version 6) with 1,000 bootstrap replicates and the branch positions of the Australian isolates from this study are indicated in red font.

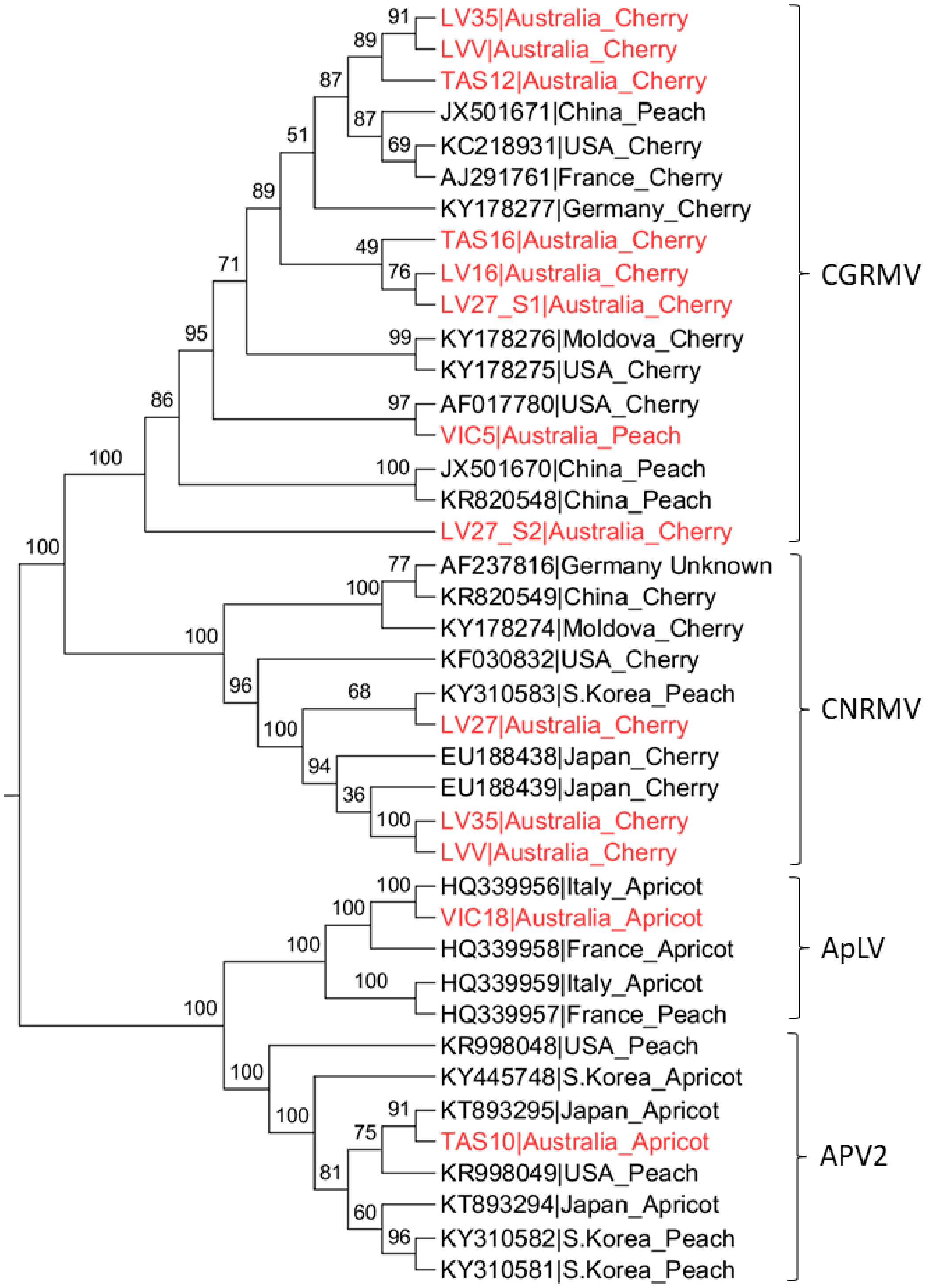

Figure S2. Maximum likelihood phylogenetic tree of full-length genome sequences of Australian *Cherry green ring mottle virus* (CGRMV), *Cherry necrotic rusty mottle virus* (CNRMV), *Apricot latent virus* (ApLV), *Asian prunus virus 2* (APV2) isolates and the corresponding GenBank isolates of each virus (Table S3). The phylogenetic tree was constructed using MEGA (version 6) with 1,000 bootstrap replicates and the branch positions of the Australian isolates from this study are indicated in red font.

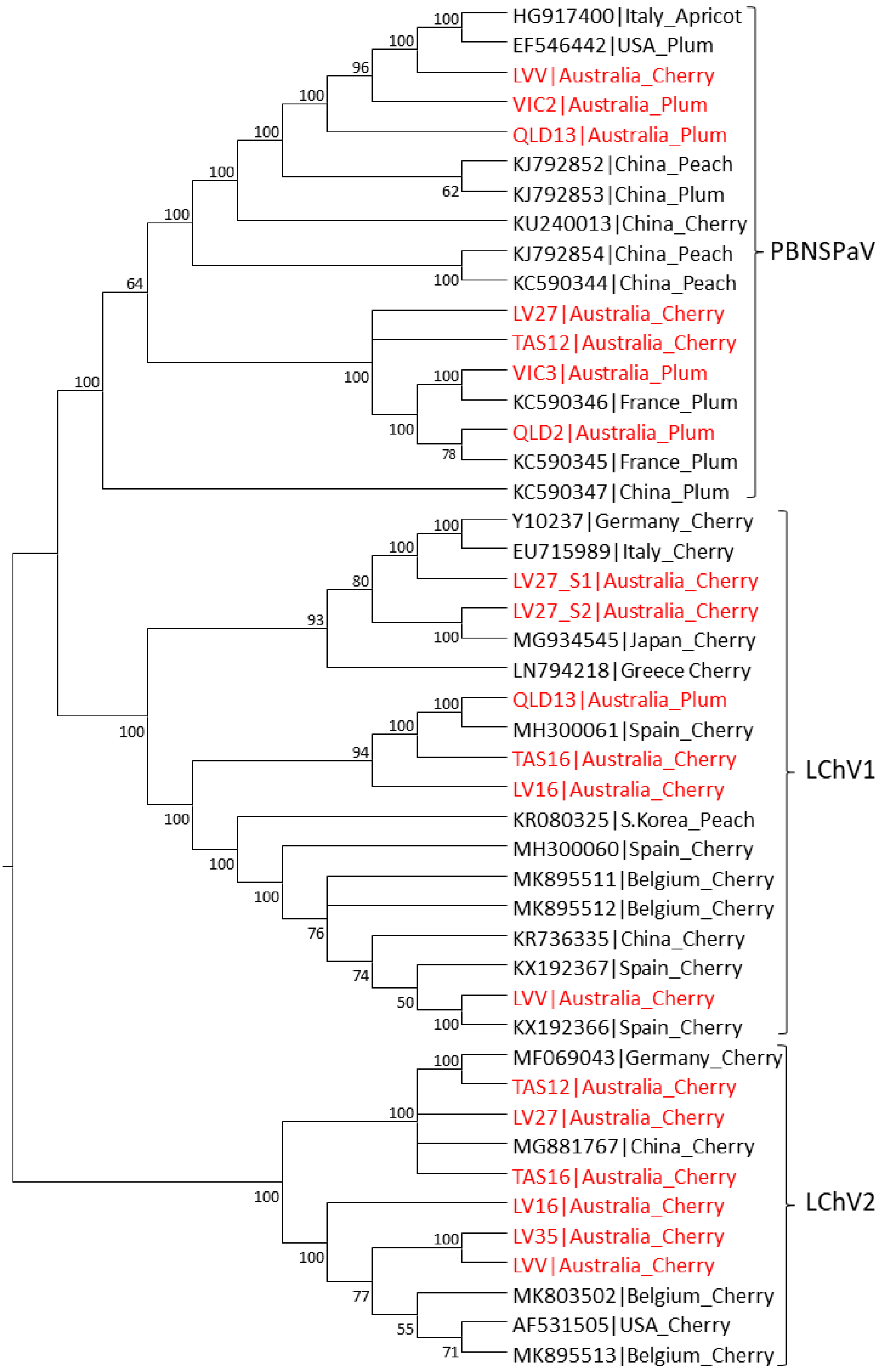

Figure S3. Maximum likelihood phylogenetic relationship of full-length genome sequences of Australian *Plum bark necrosis stem pitting-associated virus* (PBNSPaV), *Little cherry virus 1* (LChV1), *Little cherry virus 2* (LChV2) isolates and the corresponding GenBank isolates of each virus (Table S3). The phylogenetic tree was constructed using MEGA (version 6) with 1,000 bootstrap replicates and the branch positions of the Australian isolates from this study are indicated in red font.

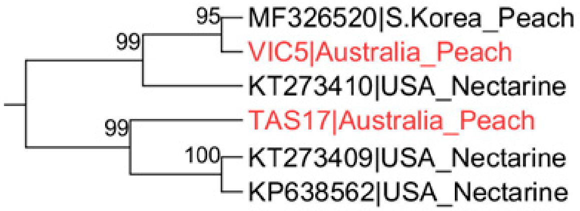

Figure S4. Maximum likelihood phylogenetic relationship of full-length genome sequences of Australian *Nectarine stem pitting-associated virus* (NSPaV) isolates and the corresponding GenBank isolates (Table S3). The phylogenetic tree was constructed using MEGA (version 6) with 1,000 bootstrap replicates and the branch positions of the Australian isolates from this study are indicated in red font.

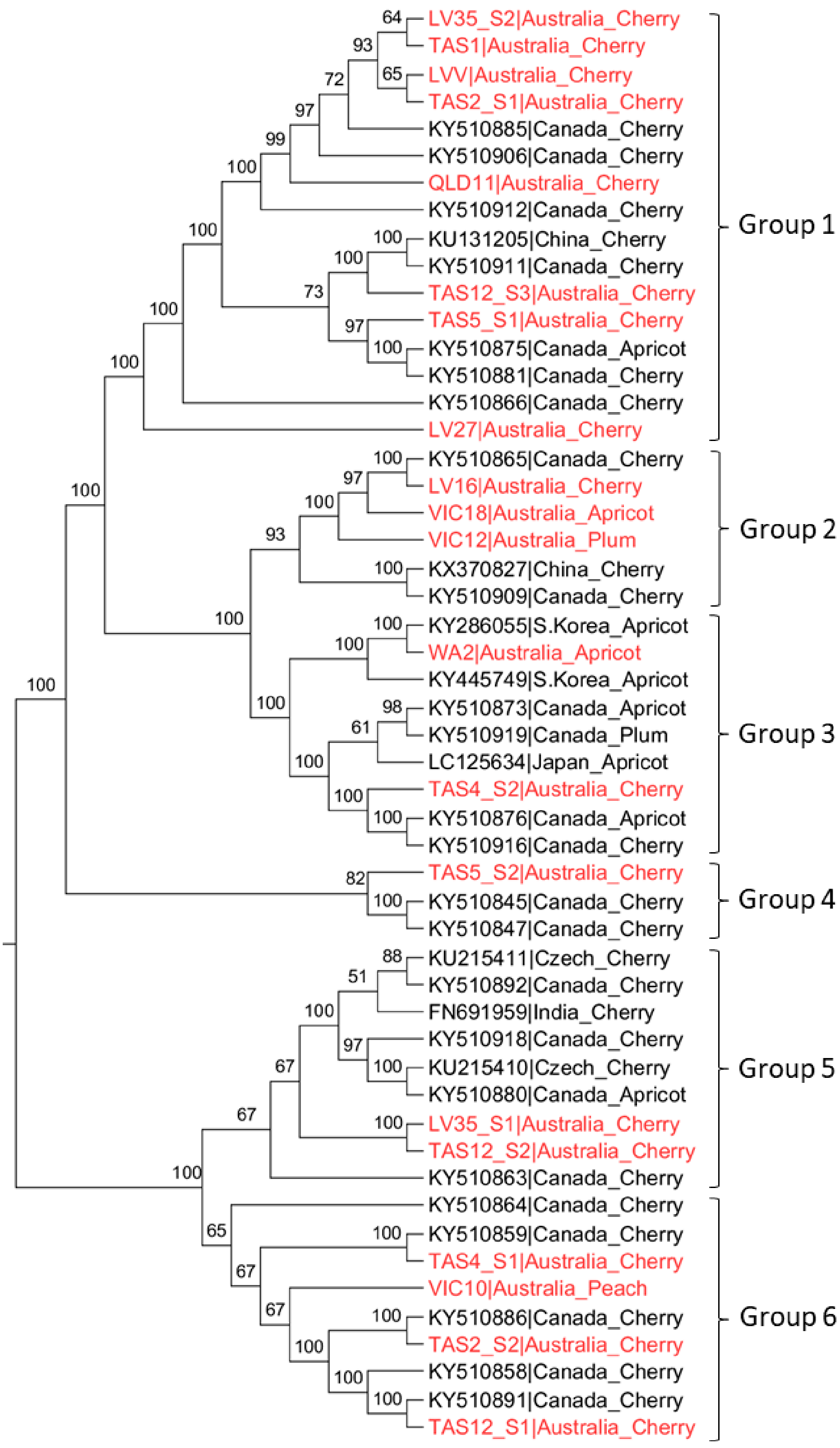

Figure S5. Maximum likelihood phylogenetic relationship of full-length genome sequences of Australian *Cherry virus A* (CVA) isolates and the corresponding GenBank isolates (Table S3). The phylogenetic tree was constructed using MEGA (version 6) with 1,000 bootstrap replicates and the branch positions of the Australian isolates from this study are indicated in red font

Table S6. The percentage (%) nucleotide identity comparison of full genome sequences of Australian isolates of *Apple chlorotic leafspot virus* (ACLSV), *Apricot latent virus* (ApLV), *Apricot pseudo-chlorotic leaf spot virus* (APCLSV), *Apricot vein clearing-associated virus* (AVCaV), *Asian prunus virus 2* (APV2), *Cherry virus A* (CVA), *Cherry green ring mottle virus* (CGRMV), *Cherry necrotic rusty mottle virus* (CNRMV), *Little cherry virus 1* (LChV1), *Little cherry virus 2* (LChV2), *Nectarine stem pitting-associated virus* (NSPaV) and *Plum bark necrosis stem pitting-associated virus* (PBNSPaV) detected in this study by metagenomic HTS and corresponding full genome sequences of GenBank isolates.

CVA	LV16	LV27	LV35_S1	LV35_S2	LVV	QLD11	TAS1	TAS12_S1	TAS12_S2	TAS12_S3	TAS2_S1	TAS2_S2	TAS4_S1	TAS4_S2	TAS5_S1	TAS5_S2	VIC10	VIC12	VIC18	WA2
LV16|Australia_Cherry	-	86%	80%	83%	83%	83%	83%	80%	80%	83%	83%	80%	81%	84%	83%	83%	81%	91%	92%	83%
LV27|Australia_Cherry	86%	-	82%	95%	94%	94%	94%	81%	82%	94%	94%	81%	81%	86%	93%	84%	82%	87%	87%	85%
LV35_S1|Australia_Cherry	80%	82%	-	81%	81%	80%	81%	81%	99%	81%	81%	81%	81%	81%	81%	81%	90%	81%	81%	81%
LV35_S2|Australia_Cherry	83%	95%	81%	-	99%	99%	99%	80%	81%	98%	100%	80%	81%	86%	98%	85%	81%	85%	85%	86%
LVV|Australia_Cherry	83%	94%	81%	99%	-	99%	99%	80%	81%	98%	100%	80%	81%	86%	98%	85%	81%	85%	85%	86%
QLD11|Australia_Cherry	83%	94%	80%	99%	99%	-	98%	80%	80%	98%	99%	80%	81%	86%	98%	85%	81%	85%	85%	86%
TAS1|Australia_Cherry	83%	94%	81%	99%	99%	98%	-	81%	81%	98%	99%	81%	81%	86%	98%	85%	81%	85%	85%	86%
TAS12_S1|Australia_Cherry	80%	81%	81%	80%	80%	80%	81%	-	81%	80%	80%	98%	86%	80%	80%	81%	89%	80%	81%	80%
TAS12_S2|Australia_Cherry	80%	82%	99%	81%	81%	80%	81%	81%	-	81%	81%	81%	81%	81%	81%	81%	90%	80%	81%	81%
TAS12_S3|Australia_Cherry	83%	94%	81%	98%	98%	98%	98%	80%	81%	-	98%	81%	81%	86%	98%	85%	81%	84%	85%	86%
TAS2_S1|Australia_Cherry	83%	94%	81%	100%	100%	99%	99%	80%	81%	98%	-	80%	81%	86%	98%	85%	81%	85%	85%	86%
TAS2_S2|Australia_Cherry	80%	81%	81%	80%	80%	80%	81%	98%	81%	81%	80%	-	86%	80%	81%	81%	90%	80%	81%	80%
TAS4_S1|Australia_Cherry	81%	81%	81%	81%	81%	81%	81%	86%	81%	81%	81%	86%	-	81%	81%	81%	85%	81%	81%	80%
TAS4_S2|Australia_Cherry	84%	86%	81%	86%	86%	86%	86%	80%	81%	86%	86%	80%	81%	-	86%	85%	81%	87%	87%	91%
TAS5_S1|Australia_Cherry	83%	93%	81%	98%	98%	98%	98%	80%	81%	98%	98%	81%	81%	86%	-	85%	81%	85%	85%	86%
TAS5_S2|Australia_Cherry	83%	84%	81%	85%	85%	85%	85%	81%	81%	85%	85%	81%	81%	85%	85%	-	81%	84%	84%	85%
VIC10|Australia_Peach	81%	82%	90%	81%	81%	81%	81%	89%	90%	81%	81%	90%	85%	81%	81%	81%	-	81%	82%	80%
VIC12|Australia_Plum	91%	87%	81%	85%	85%	85%	85%	80%	80%	84%	85%	80%	81%	87%	85%	84%	81%	-	92%	87%
VIC18|Australia_Apricot	92%	87%	81%	85%	85%	85%	85%	81%	81%	85%	85%	81%	81%	87%	85%	84%	82%	92%	-	90%
WA2|Australia_Apricot	83%	85%	81%	86%	86%	86%	86%	80%	81%	86%	86%	80%	80%	91%	86%	85%	80%	87%	90%	-
FN691959|India_Cherry	80%	82%	87%	81%	81%	81%	81%	81%	87%	81%	81%	81%	81%	81%	81%	81%	83%	81%	81%	-
KU131205|China_Cherry	83%	93%	81%	98%	98%	98%	98%	80%	81%	98%	98%	80%	81%	86%	98%	85%	81%	84%	85%	86%
KU215410|Czech_Cherry	80%	81%	87%	81%	81%	81%	81%	81%	87%	81%	81%	81%	81%	81%	81%	81%	83%	81%	81%	81%
KU215411|Czech_Cherry	81%	82%	87%	81%	81%	81%	81%	81%	87%	81%	81%	81%	81%	81%	81%	81%	83%	81%	81%	81%
KX370827|China_Cherry	83%	85%	81%	86%	86%	86%	86%	80%	81%	86%	86%	81%	81%	89%	86%	85%	81%	91%	87%	89%

KY286055|SKorea_Apricot	83%	85%	81%	86%	86%	86%	86%	80%	81%	86%	86%	80%	80%	90%	86%	85%	80%	87%	90%	99%
KY445749|SKorea_Apricot	83%	85%	81%	86%	86%	86%	86%	80%	81%	86%	86%	80%	81%	91%	86%	85%	81%	87%	91%	98%
KY510845|Canada_Cherry	82%	84%	81%	85%	85%	85%	85%	80%	81%	85%	85%	81%	82%	84%	86%	85%	81%	83%	83%	83%
KY510847|Canada_Cherry	82%	83%	81%	83%	83%	83%	83%	80%	80%	83%	83%	80%	81%	83%	83%	85%	80%	83%	83%	83%
KY510858|Canada_Cherry	80%	81%	81%	80%	80%	80%	80%	99%	81%	80%	80%	98%	86%	80%	80%	81%	89%	80%	81%	80%
KY510859|Canada_Cherry	81%	81%	81%	81%	81%	81%	81%	86%	81%	81%	81%	85%	98%	81%	81%	81%	84%	81%	81%	80%
KY510863|Canada_Cherry	80%	81%	83%	80%	80%	80%	80%	81%	83%	80%	80%	81%	80%	81%	80%	81%	82%	80%	80%	80%
KY510864|Canada_Cherry	81%	80%	82%	80%	80%	80%	80%	82%	82%	80%	80%	82%	82%	81%	80%	81%	82%	81%	81%	80%
KY510865|Canada_Cherry	99%	87%	81%	83%	83%	83%	83%	81%	81%	83%	83%	81%	81%	84%	83%	83%	81%	91%	92%	84%
KY510866|Canada_Cherry	83%	92%	81%	96%	96%	96%	96%	80%	81%	96%	96%	80%	81%	86%	96%	85%	81%	85%	85%	86%
KY510873|Canada_Apricot	84%	86%	82%	86%	86%	86%	86%	80%	81%	86%	86%	81%	81%	98%	86%	85%	81%	87%	87%	91%
KY510875|Canada_Apricot	83%	94%	81%	98%	98%	98%	98%	80%	81%	98%	98%	81%	81%	86%	98%	85%	81%	85%	85%	86%
KY510876|Canada_Apricot	84%	85%	81%	86%	86%	86%	86%	80%	81%	86%	86%	81%	81%	99%	86%	85%	81%	87%	87%	90%
KY510880|Canada_Apricot	80%	81%	87%	81%	81%	81%	81%	81%	87%	81%	81%	81%	81%	81%	81%	81%	83%	81%	81%	81%
KY510881|Canada_Cherry	83%	93%	81%	98%	98%	98%	98%	80%	81%	98%	98%	81%	81%	86%	98%	85%	81%	85%	85%	86%
KY510885|Canada_Cherry	83%	94%	81%	100%	100%	99%	99%	80%	81%	98%	100%	80%	81%	86%	98%	85%	81%	85%	85%	86%
KY510886|Canada_Cherry	80%	81%	81%	80%	80%	80%	81%	98%	81%	81%	80%	100%	86%	80%	80%	81%	90%	80%	81%	80%
KY510891|Canada_Cherry	80%	81%	81%	80%	80%	80%	81%	100%	81%	80%	80%	98%	86%	80%	80%	81%	89%	80%	81%	80%
KY510892|Canada_Cherry	81%	82%	87%	81%	81%	81%	81%	81%	87%	81%	81%	81%	81%	81%	81%	81%	83%	81%	81%	81%
KY510906|Canada_Cherry	83%	94%	81%	100%	100%	99%	99%	80%	81%	98%	100%	80%	81%	86%	98%	85%	81%	85%	85%	86%
KY510909|Canada_Cherry	83%	86%	81%	86%	86%	86%	86%	81%	81%	86%	86%	81%	81%	89%	86%	85%	81%	92%	87%	89%
KY510911|Canada_Cherry	83%	94%	81%	98%	98%	98%	98%	80%	81%	99%	98%	80%	81%	86%	98%	85%	81%	84%	85%	86%
KY510912|Canada_Cherry	83%	94%	81%	99%	99%	98%	98%	80%	81%	98%	99%	80%	81%	86%	98%	85%	81%	85%	85%	86%

KY510916|Canada_Cherry	84%	86%	81%	86%	86%	86%	86%	81%	81%	86%	86%	81%	81%	99%	86%	85%	81%	87%	87%	90%
KY510918|Canada_Cherry	80%	82%	87%	81%	81%	81%	81%	81%	87%	81%	81%	81%	81%	81%	81%	81%	83%	81%	81%	81%
KY510919|Canada_Plum	84%	86%	81%	86%	86%	86%	86%	81%	81%	86%	86%	81%	81%	98%	86%	85%	81%	87%	87%	90%
LC125634|Japan_Apricot	84%	86%	82%	86%	86%	86%	86%	80%	81%	86%	86%	80%	81%	98%	86%	85%	81%	87%	87%	90%
**CGRMV**	**TAS16**	**LV16**	**LV27_S1**	**LV27_S2**	**LV35**	**LVV**	**TAS12**	**VIC5**												
TAS16|Australia_Cherry	-	91%	95%	81%	93%	93%	93%	83%												
LV16|Australia_Cherry	91%	-	92%	82%	90%	90%	90%	83%												
LV27_S1|Australia_Cherry	95%	92%	-	81%	92%	92%	92%	83%												
LV27_S2|Australia_Cherry	81%	82%	81%	-	81%	81%	81%	80%												
LV35|Australia_Cherry	93%	90%	92%	81%	-	100%	97%	83%												
LVV|Australia_Cherry	93%	90%	92%	81%	100%	-	97%	83%												
TAS12|Australia_Cherry	93%	90%	92%	81%	97%	97%	-	83%												
VIC5|Australia_Peach	83%	83%	83%	80%	83%	83%	83%	-												
AF017780|USA_Cherry	84%	83%	83%	80%	84%	84%	84%	89%												
JX501671|China_Peach	92%	89%	91%	80%	94%	94%	94%	83%												
JX501670|China_Peach	82%	82%	82%	81%	82%	82%	82%	81%												
KY178277|Germany_Cherry	91%	89%	91%	80%	92%	92%	92%	83%												
KY178276|Moldova_Cherry	84%	83%	84%	80%	84%	84%	84%	82%												
KY178275|USA_Cherry	84%	83%	84%	80%	84%	84%	84%	82%												
KR820548|China_Peach	82%	82%	82%	81%	82%	82%	82%	81%												
KC218931|USA_Cherry	95%	90%	92%	81%	96%	96%	96%	83%												
AJ291761|France_Cherry	93%	90%	92%	81%	95%	95%	95%	83%												
**ACLSV**	**LV27**	**LV35**	**LVV**	**TAS1**	**VIC10**	**WA1**														
LV27|Australia_Cherry	-	97%	97%	97%	96%	79%														
LV35|Australia_Cherry	97%	-	97%	97%	96%	79%														
LVV|Australia_Cherry	97%	97%	-	97%	95%	79%														
TAS1|Australia_Cherry	97%	97%	97%	-	96%	79%														
VIC10|Australia_Cherry	96%	96%	95%	96%	-	79%														
WA1|Australia_Peach	79%	79%	79%	79%	79%	-														
M58152|France_Plum	79%	79%	79%	79%	79%	81%														
KX579123|Germany_Apple	79%	78%	78%	78%	78%	77%														
KX579122|Germany_Apple	77%	77%	77%	77%	77%	76%														
KU960942|China_Apple	75%	75%	75%	75%	75%	74%														
KU870525|China_Hawthorn	75%	74%	74%	74%	74%	74%														

KU870524 l China_Hawthorn	75%	75%	75%	75%	74%	75%
KC935956 l China_Pear	75%	75%	75%	75%	75%	74%
JN634761 l China_Pear	79%	79%	79%	79%	79%	81%
JN634760 l China_Pear	79%	79%	79%	79%	79%	81%
AB326225 l Japan_Apple	74%	74%	74%	74%	74%	74%
AB326224 l Japan_Apple	80%	80%	80%	80%	79%	81%
AB326223 l Japan_Apple	83%	83%	83%	83%	83%	79%
D14996 l Japan_Apple	81%	81%	81%	81%	81%	78%
MF069041 l Czech_Cherry	93%	95%	94%	94%	95%	79%
KY310578 l SKorea_Peach	79%	79%	79%	79%	79%	94%
KY310577 l SKorea_Peach	79%	79%	79%	79%	79%	81%
KY310576 l SKorea_Peach	79%	79%	79%	79%	79%	81%
KY310575 l SKorea_Peach	79%	79%	79%	79%	79%	80%
KX506849 l SKorea_Apple	83%	83%	83%	83%	83%	79%
KJ522693 l China_Apple	80%	80%	79%	80%	80%	81%
AJ243438 l Germany_Plum	80%	80%	80%	80%	79%	81%
X99752 l France_Plum	76%	76%	76%	76%	76%	75%
EU223295 l USA_Peach	68%	68%	68%	68%	68%	67%
**LChV1**	**LV16**	**LV27_S1**	**LV27_S2**	**LVV**	**QLD13**	**TAS16**
LV16 l Australia_Cherry	-	76%	77%	87%	83%	81%
LV27_S1 l Australia_Cherry	76%	-	79%	76%	76%	78%
LV27_S2 l Australia_Cherry	77%	79%	-	77%	77%	78%
LVV l Australia_Cherry	87%	76%	77%	-	77%	78%
QLD13 l Australia_Plum	83%	76%	77%	77%	-	87%
TAS16 l Australia_Cherry	81%	78%	78%	78%	87%	-
MK895512 l Belgium_Cherry	87%	76%	77%	97%	77%	78%
MK895511 l Belgium_Cherry	87%	76%	77%	97%	77%	78%
MH300061 l Spain_Cherry	89%	76%	77%	77%	90%	88%
MH300060 l Spain_Cherry	87%	76%	77%	97%	77%	78%
EU715989 l Italy_Cherry	76%	93%	77%	76%	76%	78%
MG934545 l Japan_Cherry	77%	76%	90%	77%	77%	77%
KX192367 l Spain_Cherry	87%	76%	77%	98%	77%	78%
KX192366 l Spain_Cherry	87%	76%	77%	99%	78%	78%
KR736335 l China_Cherry	87%	76%	77%	98%	77%	78%
LN794218 l Greece_Cherry	73%	73%	73%	73%	73%	72%
KR080325 l S.Korea_Peach	84%	76%	77%	90%	78%	78%
Y10237 l Germany_Cherry	76%	94%	78%	76%	76%	79%
**CNRMV**	**LV27**	**LV35**	**LVV**			
LV27 l Australia_Cherry	-	92%	92%			

LV35|Australia_Cherry	92%	-	100%																	
LVV|Australia_Cherry	92%	100%	-																	
EU188439|Japan_Cherry	91%	95%	95%																	
EU188438|Japan_Cherry	92%	95%	95%																	
KY178274|Moldova_Cherry	86%	86%	86%																	
KY310583|SKorea_Peach	94%	91%	91%																	
AF237816|Germany_NA	86%	86%	86%																	
KR820549|China_Cherry	86%	87%	86%																	
KF030832|USA_Cherry	88%	88%	88%																	
**PBNSPaV**	**LV27**	**LVV**	**QLD2**	**QLD13**	**TAS12**	**VIC2**	**VIC3**													
LV27|Australia_Cherry	-	84%	98%	84%	95%	84%	98%													
LVV|Australia_Cherry	84%	-	83%	98%	82%	98%	83%													
QLD2|Australia_Plum	98%	83%	-	83%	96%	84%	98%													
QLD13|Australia_Plum	84%	98%	83%	-	82%	98%	83%													
TAS12|Australia_Cherry	95%	82%	96%	82%	-	83%	96%													
VIC2|Australia_Plum	84%	98%	84%	98%	83%	-	83%													
VIC3|Australia_Plum	98%	83%	98%	83%	96%	83%	-													
EF546442|USA_Plum	84%	99%	83%	98%	82%	99%	83%													
KU240013|China_Cherry	84%	94%	84%	94%	83%	94%	83%													
KC590347|China_Plum	71%	71%	71%	71%	70%	71%	71%													
KC590346|France_Plum	98%	83%	99%	83%	96%	83%	99%													
KC590345|France_Plum	98%	83%	99%	83%	96%	83%	99%													
KC590344|China_Peach	84%	92%	83%	92%	82%	92%	83%													
KJ792854|China_Peach	82%	90%	82%	90%	81%	90%	82%													
KJ792853|China_Plum	83%	95%	83%	95%	82%	95%	82%													
KJ792852|China_Peach	84%	96%	83%	96%	82%	96%	83%													
HG917400|Italy_Apricot	83%	98%	82%	97%	81%	97%	82%													
**LChV2**	**LV16**	**LV27**	**LV35**	**LVV**	**TAS12**	**TAS16**														
LV16|Australia_Cherry	-	80%	87%	86%	83%	80%														
LV27|Australia_Cherry	80%	-	78%	78%	87%	87%														
LV35|Australia_Cherry	87%	78%	-	96%	78%	77%														
LVV|Australia_Cherry	86%	78%	96%	-	77%	76%														
TAS12|Australia_Cherry	83%	87%	78%	77%	-	90%														
TAS16|Australia_Cherry	80%	87%	77%	76%	90%	-														
AF531505|USA_Cherry	94%	78%	91%	90%	78%	78%														
MG881767|China_Cherry	79%	87%	77%	78%	83%	87%														
MK895513|Belgium_Cherry	91%	79%	93%	92%	79%	78%														
MK803502|Belgium_Cherry	89%	79%	93%	93%	80%	79%														
MF069043|Germany_Cherry	84%	87%	78%	77%	96%	89%														

**AVCaV**	**QLD2**	**VIC3**																		
QLD2|Australia_Plum	-	86%																		
VIC3|Australia_Nectarine	86%	-																		
KY132099|Australia_Plum	86%	99%																		
KM507062|France_Plum	87%	98%																		
HG008921|Italy_Apricot	96%	84%																		
KM507063|France_Plum	86%	98%																		
**NSPaV**	**VIC5**	**TAS17**																		
VIC5|Australia_Peach	-	94%																		
TAS17|Australia_Peach	94%	-																		
MF326520|SKorea_Peach	96%	95%																		
KT273410|USA_Nectarine	94%	94%																		
KT273409|USA_Nectarine	95%	96%																		
KP638562|USA_Nectarine	95%	96%																		
**APCLSV**	**VIC3**	**VIC11**																		
VIC3|Australia_Plum	-	96%																		
VIC11|Australia_Nectarine	96%	-																		
AY713379|Italy_Peach	81%	81%																		
KY310579|SKorea_Peach	96%	98%																		
**ApLV**	**VIC18**																			
VIC18|Australia_Apricot	-																			
HQ339959|Italy_Apricot	75%																			
HQ339958|France_Apricot	80%																			
HQ339957|France_Peach	75%																			
HQ339956|Italy_Apricot	93%																			
**APV2**	**TAS10**																			
TAS10|Australia_Apricot	-																			
KY445748|SKorea_Apricot	91%																			
KR998049|USA_Peach	94%																			
KR998048|USA_Peach	79%																			
KT893295|Japan_Apricot	96%																			
KT893294|Japan_Apricot	93%																			
KY310582|SKorea_Peach	93%																			
KY310581|SKorea_Peach	92%																			

Table S7. The recombination events identified by RDP4 package within full genome sequences of *Apple chlorotic leafspot virus* (ACLSV), *Cherry green ring mottle virus* (CGRMV), *Cherry virus A* (CVA), *Little cherry virus 1* (LChV1), *Little cherry virus 2* (LChV2) and *Plum bark necrosis stem pitting- associated virus* (PBNSPaV) isolates.

**Event**	**ACLSV isolate**	**Breakpoint position**	**Major parent**	**Minor parent**	**Genes affected**	**RDP4 detected methods**	**Highest *P* value**
1	LV27	4,251-4,625	LV35	MF069041	RdRp	R, G, B, M, C, S, 3S	5.70E-20
2	VIC10	5,020-6,414	LVV	MF069041	RdRp, MP	R, G, B, M, C, S, 3S	4.06E-17
3	VIC10	3,300-3,700	LV35	MF069041	RdRp	R, G, B, M, C, S, 3S	6.74E-18
4	LVV	1,682-1,887	VIC10	MF069041	RdRp	R, G, B, M, C, S, 3S	4.29E-13
5	LVV	2,429-2,826	LV35	MF069041	RdRp	R, G, B, M, C, S, 3S	1.51E-14
6	VIC10	4,028-4,293	LV35	MF069041	RdRp	R, G, B, M, C, S, 3S	5.29E-10
7	TAS1	4,718-5,033	LV35	MF069041	RdRp	R, G, B, C, 3S	4.42E-09
8	TAS1	3,286-3,442	LV35	MF069041	RdRp	R, G, B, M, 3S	5.55E-08
9	VIC10	843-1,008	LV35	MF069041	RdRp	R, G, B, M, S, 3S	1.24E-06
10	LV35	2,005-2,058	TAS1	MF069041	RdRp	G, B, M, C, 3S	3.45E-04
11	KX579123	6,532-7,510	M58152	D14996	CP, MP	R, G, B, M, C, S, 3S	1.36E-14
12	MF069041	255-714	LV27	LVV	Replicase	R, G, B, M, C, S, 3S	1.02E-13
13	MF069041	8-254	LVV	VIC10	Replicase	R, G, B, M, 3S	1.44E-05
**Event**	**CGRMV isolate**	**Breakpoint position**	**Major parent**	**Minor parent**	**Genes affected**	**RDP4 detected methods**	**Highest *P* value**
1	LV16	5,973-8,050	LV27_S1	LV27_S2	Hel, CP	R, G, B, M, C, S, 3S	9.37E-42
2	TAS12	796-909	LVV	AF017780	RdRp	R, G, B, M, C, 3S	1.99E-17
3	TAS16	399-1,113	LV27_S1	KC218931	RdRp	R, G, B, M, C, S	5.09E-12
4	TAS16	2,555-2,848	LV27_S1	KC218931	RdRp	R, G, B, M, C, S, 3S	9.55E-12
5	TAS16	2,022-2,235	LV27_S1	KC218931	RdRp	R, G, B, M, C, S, 3S	5.38E-05
6	TAS16	4,697-4,912	LV27_S1	KC218931	RdRp	R, G, B, M, C, S, 3S	7.52E-07
7	TAS16	5,734-5,982	LV27_S1	KC218931	RdRp	R, G, B, M, 3S	5.83E-04

8	TAS16	4,140-4,362	LV16	KC218931	RdRp	R, G, B, C,	5.71E-06
9	JX501671	7,483-8,229	TAS16	JX501670	CP	R, B, M, C, S, 3S	3.68E-29
**Event**	**CVA isolate**	**Breakpoint position**	**Major parent**	**Minor parent**	**Genes affected**	**RDP4 detected methods**	**Highest *P* value**
1	VIC10	3,839-7,350	TAS2_S2	TAS12_S2	RdRp, CP, MP	R, G, M, C, S,	6.81E-162
2	LV27	4,599-6,307	TAS2_S1	KY510865	RdRp, CP, MP	R, G, B, M, C, S, 3S	4.45E-38
3	VIC18	1,266-2,670	KY510865	KY445749	RdRp, CP, MP	R, G, M, C, S, 3S	9.47E-118
4	LV27	3,827-4,590	KY510885	LV35_S1	RdRp, CP	R, G, M, C, S, 3S	9.65E-27
5	VIC12	1,356-3,348	KY510865	KY510909	RdRp, CP	R, G, B, M, C, 3S	5.22E-63
6	VIC18	5,007-5,365	KY510865	KY445749	RdRp, CP, MP	R, G, C, 3S	8.82E-52
7	TAS1	271-419	KY510885	KY510886	RdRp, CP	R, G, C, 3S	4.29E-45
8	VIC12	1,983-2,239	KY510909	KY510865	RdRp, CP, MP	B, M, S, 3S	4.58E-36
9	VIC12	5,061-5,380	KY510865	KY510909	RdRp, CP	R, G, B, M, 3S	9.30E-33
10	VIC12	6,011-6,436	KY510865	KY510909	RdRp, CP	G, B, M, C, 3S	8.91E-27
11	QLD11	3,938-4,034	KY510912	KY510876	RdRp, CP	G, B, M, 3S	8.58E-13
12	VIC10	1-458	KY510895	LV16	RdRp, CP	R, G, M, C, S, 3S	2.56E-21
13	VIC10	2,925-3,303	KY510902	LV35_S1	RdRp, CP	R, G, M, C, S, 3S	4.88E-24
14	VIC10	504-756	KY510913	LV35_S1	RdRp, CP, MP	R, G, M, C, S, 3S	2.07E-29
15	VIC18	790-980	KY510865	KY445749	RdRp, CP	R, G, M, C, S, 3S	4.16E-07
16	VIC18	3,424-3,537	KY510865	KY445749	RdRp, CP	R, B, M, C, S, 3S	3.49E-28
17	VIC18	4,101-4,271	KY510865	KY445749	RdRp, CP	R, B, M, C, S	5.46E-04
18	KY510845	5,412-7,362	KY510879	KY510848	CP, MP, RdRp	R, G, B, M, C, S, 3S	5.61E-153
19	KY510893	6,288-7,359	KY510907	KY510909	CP, MP, RdRp	R, G, B, M, C, S, 3S	9.09E-84
20	KY510866	5,872-7,315	TAS5_S1	KY510918	CP, MP, RdRp	R, B, M, C, S, 3S	8.79E-56
21	KY510909	5,688-7,357	KY445749	KY510851	CP, MP, RdRp	R, G, B, M, C, S, 3S	5.67E-08
22	KY510909	395-593	KY510861	TAS12_S1	RdRp	R, G, B, 3S	1.53E-07
**Event**	**LChV1 isolate**	**Breakpoint position**	**Major parent**	**Minor parent**	**Genes affected**	**RDP4 detected methods**	**Highest P value**
1	LV16	8,443-16,872	QLD13	KX192367	HSP70, HP, CP	R, G, B, C, S, 3S	1.13E-06
2	TAS16	6,989-8,303	QLD13	LVV	HP	R, G, B, M, C, S, 3S	8.10E-26

3	TAS16	3,113-3,709	QLD13	Y10237	RdRp, CP	R, G, B, M, C, S, 3S	1.79E-23
4	TAS16	12,829-13,244	QLD13	KX192367	CP	R, G, B, M, C, S, 3S	7.39E-33
5	LVV	8,285-16,875	KR736335	KX192366	HSP70, HP, CP	R, G, B, M, C, S, 3S	7.86E-28
6	EU715989	15,744-16,026	Y10237	KR736335	HP	R, G, B, M, C, S	5.80E-28
7	EU715989	13,281-13,449	Y10237	KR736335	CP	R, G, B, M, C, S, 3S	4.16E-08
8	EU715989	15,672-16,160	Y10237	LV27_S1	HP	R, G, B, M, C, S	3.35E-04
**Event**	**LChV2 isolate**	**Breakpoint position**	**Major parent**	**Minor parent**	**Genes affected**	**RDP4 detected methods**	**Highest *P* value**
1	TAS16	8,562-12,198	TAS12	LV27	P53, P55, P60	R, G, B, M, C, S, 3S	8.31E-44
2	LV16	10,501-11,497	AF531505	TAS12	P53 & P60	R, G, B, M, C, S, 3S	1.65E-35
3	LV35	3,472-3,779	LVV	LV16	P 182	R, G, B, M, C, 3S	2.49E-44
4	TAS16	10,907-11,308	LV27	TAS12	P53	R, G, B, M, C, S, 3S	5.69E-33
5	LVV	3,780-3,943	LV35	LV16	P 182	R, G, B, M, C, 3S	2.21E-21
6	LVV	8,417-8,595	LV35	AF531505	P55	R, G, B, M, C, S, 3S	5.14E-23
7	TAS12	6,877-7,043	TAS16	LV27	P14	R, G, B, M, C, 3S	2.22E-05
**Event**	**PBNSPaV isolate**	**Breakpoint position**	**Major parent**	**Minor parent**	**Genes affected**	**RDP4 detected methods**	**Highest *P* value**
1	QLD2	4,519-4,817	TAS12	KJ792852	RdRp, CP	R, G, B, M, C, S, 3S	4.94E-59
2	LV27	2,172-2,548	KC590346	VIC2	RdRp, CP	R, G, B, M, C, S, 3S	1.37E-44
3	LV27	3,474-3,662	QLD2	EF546442	RdRp, CP	R, G, B, M, C, S, 3S	7.77E-28
4	TAS12	7,778-11,005	LV27	KC590345	P6	G, B, M, C, S, 3S	1.69E-13

*Coat protein (CP), heat shock (HSP), Helicase (Hel), Hypothetical protein (HP), movement protein (MP), RNA-dependent RNA polymerase (RdRp) gene.

References

1. Papademetriou, M.K.; Herath, E.; George, A.; Dorji, P.; Zailong, L. Deciduous fruit production in Asia and the Pacific. *RAP Publication (FAO)* **1999**, *10*, 38.
2. HIA. Summer fruit industry statistics. Horticulture Innovation Australia Limited: Sydney, 2016.
3. Hummer, K.E.; Janick, J. *Rosaceae*: Taxonomy, Economic Importance, Genomics. In *Genetics and Genomics of Rosaceae*, Folta, K.M., Gardiner, S.E., Eds. Springer New York: New York, NY, 2009; pp. 1-17.

4. Kloot, P. Plant introductions to South Australia prior to 1840. *Journal of the Adelaide Botanic Garden* **1985**, 217-231.
5. Quinn, G. Some notes on almonds. *Journal of Agriculture and Industry* **1904**, *7*, 335.
6. Wilkinson, J. *Almonds in Australia: from pioneer planting to prime production*; Almond Board of Australia: 2012.
7. ABA. Australian Almond Insights (Statistics). Loxton, 2019.
8. ABS. Value of Agricultural Commodities Produced in Australia. Australian Bureau of Statistics: Canberra, 2018; Vol. 7121.
9. Constable, F.; Rodoni, B. *Review of the post entry quarantine conditions for imports of almond germplasm*; DEPI: Victoria, 2011.
10. PHA. Orchard Biosecurity Manual for the Almond Industry. Plant Health Australia: Canbera, 2011.
11. Pallás, V.; Sánchez-Navarro, J.; Canizares, M. Molecular diagnostic techniques and their potential role in stone fruit certification schemes. *CIHEAM* **1998**, *19*, 191-208.
12. Gilmer, R.; Moore, J.; Nyland, G.; Welsh, M.; Pine, T.; Kirkpatrick, H.; Fulton, R. *Peach mosaic, Plum line pattern, Prunus ringspot group and Prune dwarf. Pages 50-179*; USDA, Washington, DC, 1976; Vol. 437.
13. Uyemoto, J.; Scott, S. Important diseases of *Prunus* caused by viruses and other graft-transmissible pathogens in California and South Carolina. *Plant Disease* **1992**, *76*, 5-11.
14. Nemeth, M.; Szalay-Marzsó, L.; Posnette, A. Virus, mycoplasma and rickettsia diseases of fruit trees. *Akademiai Kiado* **1986**, 256–545.
15. Tan, H.; Li, S.; Du, X.; Seno, M. First report of Cucumber mosaic virus in sweet cherry in the People's Republic of China. *Plant Disease* **2010**, *94*, 1378-1378.
16. Sato, K.; Yoshikawa, N.; Takahashi, T. Complete nucleotide sequence of the genome of an apple isolate of apple chlorotic leaf spot virus. *Journal of General Virology* **1993**, *74*, 1927-1931.
17. Marais, A.; Candresse, T.; Svanella–Dumas, L.; Jelkmann, W. Cherry virus A. *Virus and virus–like diseases of pome and stone fruits. American Phytopathological Society (APS), Minnesota* **2012**, 149-153.
18. Marais, A.; Faure, C.; Mustafayev, E.; Candresse, T. Characterization of new isolates of *Apricot vein clearing-associated virus* and of a New *Prunus*-infecting virus: Evidence for recombination as a driving force in *Betaflexiviridae* evolution. *PLoS One* **2015**, *10*, e0129469.
19. Marais, A.; Svanella-Dumas, L.; Foissac, X.; Gentit, P.; Candresse, T. Asian prunus viruses: new related members of the family Flexiviridae in Prunus germplasm of Asian origin. *Virus research* **2006**, *120*, 176-183.
20. Foissac, X.; Svanella-Dumas, L.; Dulucq, M.; Candresse, T.; Gentit, P. Polyvalent detection of fruit tree tricho, capillo and foveaviruses by nested RT-PCR using degenerated and inosine containing primers (PDO RT-PCR). *Acta Horticulturae* **2001**, *1*, 37-43.
21. Jelkmann, W. Cherry virus A: cDNA cloning of dsRNA, nucleotide sequence analysis and serology reveal a new plant capillovirus in sweet cherry. *Journal of General Virology* **1995**, *76*, 2015-2024.
22. Myrta, A.; Herranz, M.; Choueiri, E.; Pallás, V. *American Plum line pattern virus*. In *Virus and Virus-Like Diseases of Pome and Stone Fruits*, Hadidi, A., Barba, M., Candresse, T., Jelkmann, W., Eds. American Phytopathological Society: 2011; Vol. 17.
23. Villamor, D.; Eastwell, K. Viruses associated with rusty mottle and twisted leaf diseases of sweet cherry are distinct species. *Phytopathology* **2013**, *103*, 1287-1295.

24. Villamor, D.; Mekuria, T.; Pillai, S.; Eastwell, K. High-throughput sequencing identifies novel viruses in nectarine: insights to the etiology of stem-pitting disease. *Phytopathology* **2016**, *106*, 519-527.
25. Koloniuk, I.; Sarkisova, T.; Petrzik, K.; Lenz, O.; Přibylová, J.; Fránová, J.; Špak, J.; Lotos, L.; Beta, C.; Katsiani, A. Variability studies of two Prunus-infecting fabaviruses with the aid of high-throughput sequencing. *Viruses* **2018**, *10*, 204.
26. Lenz, O.; Přibylová, J.; Fránová, J.; Koloniuk, I.; Špak, J. Identification and characterization of a new member of the genus Luteovirus from cherry. *Archives of virology* **2017**, *162*, 587-590.
27. He, Y.; Cai, L.; Zhou, L.; Yang, Z.; Hong, N.; Wang, G.; Li, S.; Xu, W. Deep sequencing reveals the first fabavirus infecting peach. *Scientific reports* **2017**, *7*, 11329.
28. Maliogka, V.; Minafra, A.; Saldarelli, P.; Ruiz-García, A.; Glasa, M.; Katis, N.; Olmos, A. Recent advances on detection and characterization of fruit tree viruses using high-throughput sequencing technologies. *Viruses* **2018**, *10*, 436.
29. MacKenzie, D.J.; McLean, M.A.; Mukerji, S.; Green, M. Improved RNA extraction from woody plants for the detection of viral pathogens by reverse transcription-polymerase chain reaction. *Plant Disease Reporter* **1997**, *81* 222-226.
30. Constable, F.; Connellan, J.; Nicholas, P.; Rodoni, B. Comparison of enzyme-linked immunosorbent assays and reverse transcription-polymerase chain reaction for the reliable detection of Australian grapevine viruses in two climates during three growing seasons. *Australian Journal of Grape and Wine Research* **2012**, *18*, 239-244.
31. Thompson, J.R.; Wetzel, S.; Klerks, M.M.; Vašková, D.; Schoen, C.D.; Špak, J.; Jelkmann, W. Multiplex RT-PCR detection of four aphid-borne strawberry viruses in Fragaria spp. in combination with a plant mRNA specific internal control. *Journal of Virological Methods* **2003**, *111*, 85-93, doi:10.1016/s0166-0934(03)00164-2.
32. Nancarrow, N.; Constable, F.E.; Finlay, K.J.; Freeman, A.J.; Rodoni, B.C.; Trebicki, P.; Vassiliadis, S.; Yen, A.L.; Luck, J.E. The effect of elevated temperature on Barley yellow dwarf virus-PAV in wheat. *Virus research* **2014**, *186*, 97-103.
33. Altschul, S.F.; Madden, T.L.; Schäffer, A.A.; Zhang, J.; Zhang, Z.; Miller, W.; Lipman, D.J. Gapped BLAST and PSI-BLAST: a new generation of protein database search programs. *Nucleic Acids Research* **1997**, *25*, 3389-3402.
34. Kinoti, W.M.; Constable, F.E.; Nancarrow, N.; Plummer, K.M.; Rodoni, B. Generic Amplicon Deep Sequencing to Determine *Ilarvirus* Species Diversity in Australian *Prunus*. *Frontiers in Microbiology* **2017**, *8*, 1219.
35. Edgar, R.C. MUSCLE: multiple sequence alignment with high accuracy and high throughput. *Nucleic Acids Research* **2004**, *32*, 1792-1797.
36. Tamura, K.; Stecher, G.; Peterson, D.; Filipski, A.; Kumar, S. MEGA6: molecular evolutionary genetics analysis version 6.0. *Molecular Biology and Evolution* **2013**, *30*, 2725-2729.
37. Andrew, R. FigTree. Availabe online: http://tree.bio.ed.ac.uk/software/ (accessed on
38. Martin, D.; Rybicki, E. RDP: detection of recombination amongst aligned sequences. *Bioinformatics* **2000**, *16*, 562-563.
39. Sawyer, S. GENECONV: a computer package for the statistical detection of gene conversion. Distributed by the author, Department of Mathematics, Washington University in St. Louis. *St. Louis* **1999**.

40. Salminen, M.O.; CARR, J.K.; BURKE, D.S.; McCUTCHAN, F.E. Identification of breakpoints in intergenotypic recombinants of HIV type 1 by bootscanning. *AIDS Research and Human Retroviruses* **1995**, *11*, 1423-1425.
41. Smith, J.M. Analyzing the mosaic structure of genes. *Journal of molecular evolution* **1992**, *34*, 126-129.
42. Posada, D.; Crandall, K.A. Evaluation of methods for detecting recombination from DNA sequences: computer simulations. *Proceedings of the National Academy of Sciences* **2001**, *98*, 13757-13762.
43. Gibbs, M.J.; Armstrong, J.S.; Gibbs, A.J. Sister-scanning: a Monte Carlo procedure for assessing signals in recombinant sequences. *Bioinformatics* **2000**, *16*, 573-582.
44. Boni, M.F.; Posada, D.; Feldman, M.W. An exact nonparametric method for inferring mosaic structure in sequence triplets. *Genetics* **2007**, *176*, 1035-1047.
45. Martin, D.P.; Murrell, B.; Golden, M.; Khoosal, A.; Muhire, B. RDP4: Detection and analysis of recombination patterns in virus genomes. *Virus Evolution* **2015**, *1*.
46. Nguyen, H.D.; Tran, H.T.N.; Ohshima, K. Genetic variation of the Turnip mosaic virus population of Vietnam: a case study of founder, regional and local influences. *Virus research* **2013**, *171*, 138-149.
47. Maliogka, V.; Dovas, C.; Katis, N. Demarcation of ilarviruses based on the phylogeny of RNA2-encoded RdRp and a generic ramped annealing RT-PCR. *Archives of virology* **2007**, *152*, 1687-1698.
48. Kinoti, W.M.; Constable, F.E.; Nancarrow, N.; Plummer, K.M.; Rodoni, B. Analysis of intra-host genetic diversity of *Prunus necrotic ringspot virus* (PNRSV) using amplicon next generation sequencing. *PLoS One* **2017**, *12*.
49. Kinoti, W.; Constable, F.; Nancarrow, N.; Plummer, K.; Rodoni, B. The incidence and genetic diversity of Apple mosaic virus (ApMV) and Prune dwarf virus (PDV) in Prunus species in Australia. *Viruses* **2018**, *10*, 136.
50. Stubs, L.; Smith, P. The association of Prunus ringspot, prune dwarf, and dark green sunken mottle viruses in the rosetting and decline disease of peach. *Crop and Pasture Science* **1971**, *22*, 771-785.
51. Revill, P.A. *Operations Manual for the Fruit Variety Foundation (FVF) of Australia, 1988*; Department of Agriculture and Rural Affairs: 1989.
52. Mekuria, T.; Smith, T.; Beers, E.; Watson, G.; Eastwell, K. First report of transmission of Little cherry virus 2 to sweet cherry by Pseudococcus maritimus (Ehrhorn)(Hemiptera: Pseudococcidae). *Plant Disease* **2013**, *97*, 851-851.
53. Pallas, V.; Aparicio, F.; Herranz, M.; Amari, K.; Sanchez-Pina, M.; Myrta, A.; Sanchez-Navarro, J. Ilarviruses of *Prunus* spp.: A continued concern for fruit trees. *Phytopathology* **2012**, *102*, 1108-1120.
54. Greber, R.; Klose, M.; Milne, J.; Teakle, D. Transmission of prunus necrotic ringspot virus using plum pollen and thrips. *Annals of Applied Biology* **1991**, *118*, 589-593.
55. Greber, R.; Teakle, D.; Mink, G. Thrips-facilitated transmission of *Prune dwarf* and *Prunus necrotic ringspot viruses* from cherry pollen to cucumber. *Plant Disease* **1992**, *76*, 1039-1041.
56. Gilmer, R. Host range and variable pathogenesis of the necrotic ring spot virus in the genus *Prunus*. *Plant Disease Reporter* **1955**, *39*, 194-201.
57. Cropley, R.; Gilmer, R.; Posnette, A. Necrotic ring spot and prune dwarf viruses in *Prunus* and in herbaceous indicators. *Annals of Applied Biology* **1964**, *53*, 325-332.
58. Chandel, V.; Rana, T.; Handa, A.; Thakur, P.; Hallan, V.; Zaidi, A. Incidence of *Prunus necrotic ringspot virus* on *Malus domestica* in India. *Journal of Phytopathology* **2008**, *156*, 382-384.
59. Dhir, S.; Tomar, M.; Thakur, P.; Ram, R.; Hallan, V.; Zaidi, A. Molecular evidence for Apple stem pitting virus infection in India. *Plant Pathology* **2010**, *59*.

60. Yang, H.; Liu, Z.; Luo, S.; Li, L. First Report of Apple stem pitting virus Infecting Nanking Cherry in China. *Plant Disease* **2017**, *101*, 1067.
61. Yu, C.-M.; Geng, G.-W.; Cao, X.-R.; Yang, C.; Qi, Z.; Liu, S.-S.; Zhu, C.-X.; Yuan, X.-F. First identification of cucumber mosaic virus infecting six fruit crops in China. *Journal of Plant Pathology* **2019**, *101*, 373-376.
62. Negi, A.; Rana, T.; Kumar, Y.; Ram, R.; Hallan, V.; Zaidi, A.A. Analysis of the coat protein gene of Indian strain of Apple stem grooving virus. *Journal of plant biochemistry and biotechnology* **2010**, *19*, 91-94.
63. Constable, F.E.; Joyce, P.A.; Rodoni, B.C. A survey of key Australian pome fruit growing districts for exotic and endemic pathogens. *Australasian Plant Pathology* **2007**, *36*, 165-172, doi:10.1071/ap07003.
64. Roistacher, C.; Nauer, E.; Wagner, R. Transmissibility off Cachexia, Dweet Mottle, Psorosis, Tatterleaf and Infectious Variegation Viruses on Knife Blades and Its Prevention. In Proceedings of International Organization of Citrus Virologists Conference Proceedings (1957-2010).
65. Bag, S.; Al Rwahnih, M.; Li, A.; Gonzalez, A.; Rowhani, A.; Uyemoto, J.K.; Sudarshana, M.R. Detection of a new luteovirus in imported nectarine trees: a case study to propose adoption of metagenomics in post-entry quarantine. *Phytopathology* **2015**, *105*, 840-846.
66. Smith, P. The Australian Fruit Variety Foundation and its role in supplying virus-tested planting material to the fruit industry. In Proceedings of XII International Symposium on Fruit Tree Virus Diseases 130; pp. 263-266.
67. Aparicio, F.; Sánchez-Pina, M.A.; Sánchez-Navarro, J.A.; Pallás, V. Location of *Prunus necrotic ringspot ilarvirus* within pollen grains of infected nectarine trees: evidence from RT-PCR, dot-blot and in situ hybridisation. *European Journal of Plant Pathology* **1999**, *105*, 623-627.
68. Fulton, R. *Prunus necrotic ringspot virus*. *CMI/AAB descriptions of plant viruses* **1970**, *5*.
69. Zong, X.; Wang, W.; Wei, H.; Wang, J.; Yan, X.; Hammond, R.; Liu, Q. Incidence of sweet cherry viruses in Shandong Province, China and a case study on multiple infection with five viruses. *Journal of Plant Pathology* **2015**, *97*, 61-68.
70. Myrta, A.; Di Terlizzi, B.; Savino, V.; Martelli, G. Virus diseases affecting the Mediterranean stone fruit industry: a decade of surveys. *Virus and virus-like diseases of stone fruits, with particular reference to the Mediterranean region. Bari: CIHEAM (Centre International de Hautes Etudes Agronomiques Méditerranéennes) Options Méditerranéennes, Série B* **2003**, 15-23.
71. Xu, Y.; Li, S.; Na, C.; Yang, L.; Lu, M. Analyses of virus/viroid communities in nectarine trees by next-generation sequencing and insight into viral synergisms implication in host disease symptoms. *Scientific reports* **2019**, *9*, 1-12.
72. Tahzima, R.; Foucart, Y.; Peusens, G.; Beliën, T.; Massart, S.; De Jonghe, K. High-Throughput Sequencing Assists Studies in Genomic Variability and Epidemiology of Little Cherry Virus 1 and 2 infecting Prunus spp. in Belgium. *Viruses* **2019**, *11*, 592.
73. Cañizares, M.C.; Marcos, J.F.; Pallás, V. Molecular characterization of an almond isolate of hop stunt viroid (HSVd) and conditions for eliminating spurious hybridization in its diagnosis in almond samples. *European Journal of Plant Pathology* **1999**, *105*, 553-558.
74. Katsiani, A.; Maliogka, V.; Candresse, T.; Katis, N. Host-range studies, genetic diversity and evolutionary relationships of ACLSV isolates from ornamental, wild and cultivated R osaceous species. *Plant Pathology* **2014**, *63*, 63-71.

75. Liberti, D.; Marais, A.; Svanella-Dumas, L.; Dulucq, M.; Alioto, D.; Ragozzino, A.; Rodoni, B.; Candresse, T. Characterization of Apricot pseudo-chlorotic leaf spot virus, a novel Trichovirus isolated from stone fruit trees. *Phytopathology* **2005**, *95*, 420-426.
76. Al Rwahnih, M.; Turturo, C.; Minafra, A.; Saldarelli, P.; Myrta, A.; Pallás, V.; Savino, V. Molecular variability of Apple chlorotic leaf spot virus in different hosts and geographical regions. *Journal of Plant Pathology* **2004**, 117-122.
77. Osman, F.; Al Rwahnih, M.; Golino, D.; Pitman, T.; Cordero, F.; Preece, J.; Rowhani, A. Evaluation of the phytosanitary status of the Prunus species in the national clonal germplasm repository in California: survey of viruses and viroids. *Journal of Plant Pathology* **2012**, *94*, 249-253.
78. Candresse, T.; Lanneau, M.; Revers, F.; Macquaire, G.; German, S.; Dunez, J.; Grasseau, N.; Malinovsky, T. An immunocapture PCR assay adapted to the detection and the analysis of the molecular variability of apple chlorotic leaf spot virus. In Proceedings of XVI International Symposium on Fruit Tree Virus diseases 386; pp. 136-147.
79. Liberti, D.; Ragozzino, A.; Gentit, P.; Marais, A.; Svanella-Dumas, L.; Candresse, T. Biological properties and partial molecular characterization of an apricot strain of CGRMV. In Proceedings of XIX International Symposium on Virus and Virus-like Diseases of Temperate Fruit Crops-Fruit Tree Diseases 657; pp. 103-108.
80. Rott, M.; Xiang, Y.; Boyes, I.; Belton, M.; Saeed, H.; Kesanakurti, P.; Hayes, S.; Lawrence, T.; Birch, C.; Bhagwat, B. Application of next generation sequencing for diagnostic testing of tree fruit viruses and viroids. *Plant Disease* **2017**, *101*, 1489-1499.
81. Blouin, A.G.; Ross, H.A.; Hobson-Peters, J.; O'Brien, C.A.; Warren, B.; MacDiarmid, R. A new virus discovered by immunocapture of double-stranded RNA, a rapid method for virus enrichment in metagenomic studies. *Molecular Ecology Resources* **2016**, *5*, 1255-1263.
82. Qu, L.; Cui, H.; Wu, G.; Zhou, J.; Su, J.; Wang, G.; Hong, N. Genetic diversity and molecular evolution of Plum bark necrosis stem pitting-associated virus from China. *PLoS One* **2014**, *9*, e105443.
83. Al Rwahnih, M.; Uyemoto, J.; Falk, B.; Rowhani, A. Molecular characterization and detection of plum bark necrosis stem pitting-associated virus. *Archives of virology* **2007**, *152*, 2197-2206.
84. Gao, R.; Xu, Y.; Candresse, T.; He, Z.; Li, S.; Ma, Y.; Lu, M. Further insight into genetic variation and haplotype diversity of Cherry virus A from China. *PLoS One* **2017**, *12*, e0186273.
85. Marais, A.; Faure, C.; Candresse, T. New insights into Asian *Prunus* viruses in the light of NGS-based full genome sequencing. *PLoS One* **2016**, *11*, e0146420, doi:10.1371/journal.pone.0146420.
86. Kesanakurti, P.; Belton, M.; Saeed, H.; Rast, H.; Boyes, I.; Rott, M. Comparative analysis of cherry virus A genome sequences assembled from deep sequencing data. *Archives of virology* **2017**, *162*, 2821-2828.
87. Glasa, M.; Šoltys, K.; Vozárová, Z.; Predajňa, L.; Sihelská, N.; Šubr, Z.; Candresse, T. High intra-host cherry virus a population heterogeneity in cherry trees in Slovakia. *Journal of Plant Pathology* **2017**, *99*, 745-752.
88. Simon, A.; Bujarski, J. RNA-RNA recombination and evolution in virus-infected plants. *Annual Review of Phytopathology* **1994**, *32*, 337-362.
89. Worobey, M.; Holmes, E.C. Evolutionary aspects of recombination in RNA viruses. *Journal of General Virology* **1999**, *80*, 2535-2543.
90. Katsiani, A.; Maliogka, V.; Amoutzias, G.; Efthimiou, K.; Katis, N. Insights into the genetic diversity and evolution of L ittle cherry virus 1. *Plant Pathology* **2015**, *64*, 817-824.

91. Chen, S.; Zhou, Y.; Ye, T.; Hao, L.; Guo, L.; Fan, Z.; Li, S.; Zhou, T. Genetic variation analysis of apple chlorotic leaf spot virus coat protein reveals a new phylogenetic type and two recombinants in China. *Archives of virology* **2014**, *159*, 1431-1438.
92. Marais, A.; Faure, C.; Mustafayev, E.; Barone, M.; Alioto, D.; Candresse, T. Characterization by deep sequencing of Prunus virus T, a novel Tepovirus infecting Prunus species. *Phytopathology* **2015**, *105*, 135-140.
93. James, D.; Phelan, J.; Sanderson, D. Detection by high-throughput sequencing and molecular characterization of complexes of fabaviruses infecting 'Staccato (R)'sweet cherry (Prunus avium) in Canada. *Canadian Journal of Plant Pathology* **2019**, 1-16.
94. Hou, Q.; Han, T.; Li, L.; Wang, J.; Yu, M.; Zhang, S.; Cao, M.; Yang, C. The complete nucleotide sequence and genome organization of a novel virus of the order Tymovirales isolated from Prunus davidiana (Carr.) Franch. in Liaoning, China. *Archives of virology* **2019**, *164*, 1245-1248.
95. Barba, M.; Czosnek, H.; Hadidi, A. Historical perspective, development and applications of next-generation sequencing in plant virology. *Viruses* **2014**, *6*, 106-136.
96. Massart, S.; Olmos, A.; Jijakli, H.; Candresse, T. Current impact and future directions of high throughput sequencing in plant virus diagnostics. *Virus research* **2014**, *188*, 90-96.
97. Candresse, T.; Filloux, D.; Muhire, B.; Julian, C.; Galzi, S.; Fort, G.; Bernardo, P.; Daugrois, J.-H.; Fernandez, E.; Martin, D.P. Appearances can be deceptive: revealing a hidden viral infection with deep sequencing in a plant quarantine context. *PLoS One* **2014**, *9*, e102945.
98. Sanchez-Navarro, J.; Aparicio, F.; Herranz, M.; Minafra, A.; Myrta, A.; Pallas, V. Simultaneous detection and identification of eight stone fruit viruses by one-step RT-PCR. *European Journal of Plant Pathology* **2005**, *111*, 77-84.
99. Petrzik, K.; Svoboda, P. Screening of *Apple mosaic virus* in hop cultivars in the Czech Republic by reverse transcription-polymerase chain reaction. *Acta virologica* **1997**, *41*, 101-103.
100. Ito, T.; Ieki, H.; Ozaki, K. Simultaneous detection of six citrus viroids and Apple stem grooving virus from citrus plants by multiplex reverse transcription polymerase chain reaction. *Journal of Virological Methods* **2002**, *106*, 235-239.
101. Menzel, W.; Jelkmann, W.; Maiss, E. Detection of four apple viruses by multiplex RT-PCR assays with coamplification of plant mRNA as internal control. *Journal of Virological Methods* **2002**, *99*, 81-92.
102. García-Ibarra, A.; Martinez-Gomez, P.; Rubio, M.; Dicenta, F.; Soler, A.; Pallas, V.; Sanchez-Navarro, J. First report of Apricot latent virus and Plum bark necrosis stem pitting-associated virus in apricot from Spain. *Plant Disease* **2010**, *94*, 275-275.
103. Li, R.; Mock, R. An improved reverse transcription-polymerase chain reaction (RT-PCR) assay for the detection of two cherry flexiviruses in Prunus spp. *Journal of Virological Methods* **2005**, *129*, 162-169.
104. Wylie, S.; Wilson, C.; Jones, R.; Jones, M. A polymerase chain reaction assay for cucumber mosaic virus in lupin seeds. *Australian Journal of Agricultural Research* **1993**, *44*, 41-51.
105. Rott, M.; Jelkmann, W. Characterization and detection of several filamentous viruses of cherry: adaptation of an alternative cloning method (DOP-PCR), and modification of an RNA extraction protocol. *European Journal of Plant Pathology* **2001**, *107*, 411-420.
106. Jelkmann, W.; Leible, S.; Rott, M. Little cherry closteroviruses-1 and-2, their genetic variability and detection by Real-Time-PCR. In Proceedings of XX International Symposium on Virus and Virus-Like Diseases of Temperate Fruit Crops-Fruit Tree Diseases 781; pp. 321-330.

107. Ghanem-Sabanadzovic, N.A.; Mahboubi, M.; Di Terlizzi, B.; Sabanadzovic, S.; Savino, V.; Uyemoto, J.; Martelli, G. Molecular detection of a closterovirus associated with apricot stem pitting in Southern Italy. *Journal of Plant Pathology* **2001**, *83*, 125-132.
108. Parakh, D.; Shamloul, A.; Hadidi, A.; Waterworth, H.; Scott, S.; Howell, H.; Mink, G. Detection of *Prune dwarf ilarvirus* from infected stone fruits using reverse transcription-polymerase chain reaction. *Acta Horticulturae* **1994**, *386*, 421-430.
109. Faggioli, F.; Ragozzino, E. Detection of pome fruit viroids by RT-PCR using a single primer pair. *Journal of Plant Pathology* **2002**, 125-128.
110. Wetzel, T.; Jardak, R.; Meunier, L.; Ghorbel, A.; Reustle, G.; Krczal, G. Simultaneous RT/PCR detection and differentiation of arabis mosaic and grapevine fanleaf nepoviruses in grapevines with a single pair of primers. *Journal of Virological Methods* **2002**, *101*, 63-69.
111. Werner, R.; Mühlbach, H.P.; Büttner, C. Detection of cherry leaf roll nepovirus (CLRV) in birch, beech and petunia by immuno-capture RT-PCR using a conserved primer pair. *European Journal of Forest Pathology* **1997**, *27*, 309-318.
112. James, D.; Upton, C. Single primer pair designs that facilitate simultaneous detection and differentiation of peach mosaic virus and cherry mottle leaf virus. *Journal of Virological Methods* **1999**, *83*, 103-111.
113. James, D.; Howell, W.; Mink, G. Molecular evidence of the relationship between a virus associated with flat apple disease and Cherry rasp leaf virus as determined by RT-PCR. *Plant Disease* **2001**, *85*, 47-52.
114. Domingo, E.; Martinez-salas, E.; Sobrino, F.; Carlos, J.; Tortea, D.; Ortin, J.; Lpez-galindezb, C.; Pkez-breaab, P.; Villanuevab, N.; Nhjera, R. The quasispecies nature of viral RNA genome populations : biological relevance. *Gene* **1985**, *40*.
115. Shamloul, A.; Minafra, A.; Hadidi, A.; Waterworth, H.; Giunchedi, L.; Allam, E. Peach latent mosaic viroid: nucleotide sequence of an Italian isolate, sensitive detection using RT-PCR and geographic distribution. In Proceedings of XVI International Symposium on Fruit Tree Virus diseases 386; pp. 522-530.
116. Ambrós, S.; Hernández, C.; Desvignes, J.; Flores, R. Genomic structure of three phenotypically different isolates of peach latent mosaic viroid: implications of the existence of constraints limiting the heterogeneity of viroid quasispecies. *Journal of virology* **1998**, *72*, 7397-7406.
117. James, D.; Varga, A.; Pallas, V.; Candresse, T. Strategies for simultaneous detection of multiple plant viruses. *Canadian Journal of Plant Pathology* **2006**, *28*, 16-29.
118. Lebas, B.; Ward, L. Vitis (grapevine) post-entry quarantine testing manual. *Plant health and environment laboratory, Ministry for Primary Industries, New Zealand* **2012**, 1-2.
119. Wetzel, T.; Candresse, T.; Ravelonandro, M.; Dunez, J. A polymerase chain reaction assay adapted to plum pox potyvirus detection. *Journal of Virological Methods* **1991**, *33*, 355-365.
120. Fuchs, M.; Abawi, G.; Marsella-Herrick, P.; Cox, R.; Cox, K.; Carroll, J.; Martin, R. Occurrence of Tomato ringspot virus and Tobacco ringspot virus in highbush blueberry in New York State. *Journal of Plant Pathology* **2010**, 451-459.
121. Harper, S.J.; Delmiglio, C.; Ward, L.I.; Clover, G.R. Detection of Tomato black ring virus by real-time one-step RT-PCR. *Journal of Virological Methods* **2011**, *171*, 190-194.
122. Russo, M.; Vovlas, C.; Rubino, L.; Grieco, F.; Martelli, G. Molecular characterization of a tombusvirus isolated from diseased pear trees in southern Italy. *Journal of Plant Pathology* **2002**, 161-166.

123. Griesbach, J.A. Detection of tomato ringspot virus by polymerase chain reaction. *Plant disease (USA)* **1995**.
124. Dovas, C.; Katis, N. A spot nested RT-PCR method for the simultaneous detection of members of the Vitivirus and Foveavirus genera in grapevine. *Journal of Virological Methods* **2003**, *107*, 99-106.
125. Dovas, C.; Katis, N. A spot multiplex nested RT-PCR for the simultaneous and generic detection of viruses involved in the aetiology of grapevine leafroll and rugose wood of grapevine. *Journal of Virological Methods* **2003**, *109*, 217-226.
